# Cerebral Intraparenchymal Hemorrhage due to Implantation of Electrodes for Deep Brain Stimulation: Insights from a Large Single-Center Retrospective Cross-Sectional Analysis

**DOI:** 10.3390/brainsci14060612

**Published:** 2024-06-19

**Authors:** Bastian E. A. Sajonz, Timo S. Brugger, Marco Reisert, Martin Büchsel, Nils Schröter, Alexander Rau, Karl Egger, Peter C. Reinacher, Horst Urbach, Volker A. Coenen, Christoph P. Kaller

**Affiliations:** 1 Department of Stereotactic and Functional Neurosurgery, Medical Center — University of Freiburg, Faculty of Medicine, University of Freiburg, 79106 Freiburg, Germany; 2 Department of Neuroradiology, Medical Center — University of Freiburg, Faculty of Medicine, University of Freiburg, 79106 Freiburg, Germany; 3 Institute for Evidence in Medicine, Medical Center — University of Freiburg, Faculty of Medicine, University of Freiburg, 79110 Freiburg, Germany; 4 Cochrane Germany, Cochrane Germany Foundation, 79110 Freiburg, Germany; 5 Medical Physics, Department of Radiology, Medical Center — University of Freiburg, Faculty of Medicine, University of Freiburg, 79106 Freiburg, Germany; 6 Institute of Clinical Chemistry and Laboratory Medicine, Medical Center — University of Freiburg, Faculty of Medicine, University of Freiburg, 79106 Freiburg, Germany; 7 Department of Neurology, Medical Center — University of Freiburg, Faculty of Medicine, University of Freiburg, 79106 Freiburg, Germany; 8 Department of Radiology, Tauernklinikum, 5700 Zell am See, Austria; 9 Paracelsus Medical Private University (PMU), 5020 Salzburg, Austria; 10 Fraunhofer Institute for Laser Technology (ILT), 52074 Aachen, Germany; 11 Center for Deep Brain Stimulation, University of Freiburg, 79106 Freiburg, Germany; 12 Freiburg Optical NeuroImaging [FrONI], Medical Center — University of Freiburg, Faculty of Medicine, University of Freiburg, 79106 Freiburg, Germany

**Keywords:** cerebral hemorrhage, deep brain stimulation, risk factors, aged, microelectrodes, retrospective studies

## Abstract

Cerebral intraparenchymal hemorrhage due to electrode implantation (CIPHEI) is a rare but serious complication of deep brain stimulation (DBS) surgery. This study retrospectively investigated a large single-center cohort of DBS implantations to calculate the frequency of CIPHEI and identify patient- and procedure-related risk factors for CIPHEI and their potential interactions. We analyzed all DBS implantations between January 2013 and December 2021 in a generalized linear model for binomial responses using bias reduction to account for sparse sampling of CIPHEIs. As potential risk factors, we considered age, gender, history of arterial hypertension, level of invasivity, types of micro/macroelectrodes, and implanted DBS electrodes. If available, postoperative coagulation and platelet function were exploratorily assessed in CIPHEI patients. We identified 17 CIPHEI cases across 839 electrode implantations in 435 included procedures in 418 patients (3.9%). Exploration and cross-validation analyses revealed that the three-way interaction of older age (above 60 years), high invasivity (i.e., use of combined micro/macroelectrodes), and implantation of directional DBS electrodes accounted for 82.4% of the CIPHEI cases. Acquired platelet dysfunction was present only in one CIPHEI case. The findings at our center suggested implantation of directional DBS electrodes as a new potential risk factor, while known risks of older age and high invasivity were confirmed. However, CIPHEI risk is not driven by the three factors alone but by their combined presence. The contributions of the three factors to CIPHEI are hence not independent, suggesting that potentially modifiable procedural risks should be carefully evaluated when planning DBS surgery in patients at risk.

## 1. Introduction

Deep brain stimulation (DBS) improves symptoms and quality of life in pharmacotherapy-resistant neurological diseases, such as Parkinson’s disease (PD), tremor, dystonia, epilepsy, and pain (cf. [1]). DBS has also emerged as an investigational treatment for Alzheimer’s disease and therapy-resistant psychiatric conditions and was even approved for obsessive-compulsive disorder in the past [1].

Adverse events following DBS implantation are rare [2]. Cerebral intraparenchymal hemorrhage due to electrode implantation (CIPHEI) is the most serious complication. Estimates of the frequency of hemorrhage due to electrode implantation range between 2 and 5% per patient [2,3,4,5,6,7], with symptomatic hemorrhages, permanent deficits, and death in up to 2.1%, 1.7%, and 0.2%, respectively.

Patient-related CIPHEI risk factors comprise older age [3,8,9,10,11,12], male gender [12,13], and a history of hypertension/intraoperatively elevated blood pressure [3,8,9,13,14,15]. Associations with the diagnosis of PD [11,12] and specific DBS targets [14,16] were also reported. Microelectrode recording (MER: use of combined micro/macroelectrodes (CMM) with extended microelectrodes) for target verification constitutes the most relevant procedure-related risk factor [3,5]. Efforts to minimize invasivity include reduction of microelectrode passes [17], different microelectrode designs [10], or abandonment of MER [3]. However, MER provides functional information from the target to verify correct DBS implantation [18], in particular as DBS surgery is increasingly performed under general anesthesia [19].

The assessment of patient- and procedure-related CIPHEI risk factors faces several challenges. First, disentangling the contributions of individual risk factors is hampered by confounding between variables, e.g., specific combinations of brain targets and diagnoses are naturally associated with specific patient-related characteristics and procedural approaches. This requires a multivariable approach, whereas (commonly applied) bivariable analysis may lead to invalid findings and misinterpretations [20].

Second, CIPHEI constitutes a rare phenomenon, which may bias the analysis of their true frequency and the relevance of potential risk factors if sparse sampling is not addressed [21,22].

Third, hemorrhagic events are categorized differently across the literature (e.g., intracranial vs. intracerebral hemorrhages with/without further differentiation into intraparenchymal/intraventricular), impeding comparisons. Previous studies report mainly on intracranial/intracerebral hemorrhages, with little or no further differentiation [2,5,6,7,9,11,23,24,25,26,27], and only Fenoy and Simpson [28] specified intracerebral vs. intraventricular origins.

Fourth, although MER is a known CIPHEI risk factor, the role of the inserted guiding cannula versus the actually extended microelectrodes remains unclear, with only Ben-Haim et al. [10] providing this degree of differentiation.

The objective of this retrospective cross-sectional analysis was to present the frequency of CIPHEI across the sample and to identify potential risk factors and the nature of their interrelation, while addressing the methodical issues listed above. Considering the multitude of unmodifiable patient-related factors, a key question is whether potentially modifiable procedural choices may balance a patient’s overall risk. For this purpose, it is, however, crucial to know if factors contribute independently or if the presence of one factor may potentiate (or alleviate) the risk associated with the presence of another factor.

## 2. Methods

In this single-center retrospective cross-sectional study, all consecutive patients were included in the screening who received DBS electrode implantation under the current surgical team (January 2013 to December 2021) at the Department of Stereotactic and Functional Neurosurgery of the Medical Center — University of Freiburg. There was no exclusion criterion for the screening. The study was approved by the local institutional review board. Informed written consent was waived due to the study’s retrospective nature. Computed tomography (CT) imaging and clinical and demographic data were reviewed and, if available, postoperative analyses of coagulation and platelet function in CIPHEI cases.

DBS electrode implantation was performed according to institutional standards provided in Supplementary Materials S1.

CIPHEI comprised the target outcome. All diagnoses of intracranial hemorrhages were based on evaluation of postoperative CT scans by board-certified neuroradiologists. Intracranial but extra-parenchymal hemorrhages (subdural, subarachnoidal, and intraventricular origin) were descriptively reported (Supplementary Materials S2) but not considered in our analyses since these generally constitute small and self-limiting bleeding events without clinically apparent sequelae.

Following Ben-Haim et al. [10], CIPHEIs were further differentiated into deep bleedings (≤25 mm above the tip of the DBS electrode) to identify CIPHEI that originated from the CMM (vs. those with mere contact with the guiding cannulas).

Patient-related factors comprised age, gender, and history of arterial hypertension. Procedure-related factors concerned the level of invasivity (use of CMM with vs. without MER vs. blunt-tip electrode) and types of CMM and DBS electrodes (see Table 1).

The diagnosis treated for and the target for DBS were originally considered as factors of interest but were omitted over the course of the analysis to handle the substantial confounding with other patient- and procedure-related factors (Supplementary Materials S3).

With respect to the level of invasivity, the use of blunt-tip electrodes was regarded as low invasive, while the use of CMM was regarded as medium invasive with retracted microelectrodes (which leaves them still more sharp-edged than a blunt-tip electrode) and as high invasive with microelectrodes extended for MER.

Analysis of coagulation and platelet function is detailed in Supplementary Materials S4.

Considering the dichotomous nature of CIPHEI (yes/no), analyses were based on generalized linear models for binomial responses using the bias-reduction approach (BR-GLM) developed by Firth [21] to account for sparse sampling. Analyses hence corresponded to logistic regressions with bias removal from the maximum likelihood estimator [22] and were conducted in R statistical software (version 4.2.2 [29]) using the brglm package (version 0.7.2 [30]). Significance and trends thereof were assessed at an alpha level of *p* = 0.05 and *p* = 0.10, respectively.

Bivariable analyses are reported for comparability with previous studies, whereas identification of potential risk factors in the present study was based on multivariable analyses considering the potential issues of invalid findings associated with bivariable analyses in light of confounding [20]. Model selection and stepwise backward elimination of (non-significant) terms were based on the Akaike Information Criterion (AIC [31]), which weights the model’s goodness of fit and complexity and is asymptotically valid for BR-GLMs [30].

Note that for analysis of CIPHEI at any point of the trajectory, medium/high invasivity (the use of CMM irrespective of MER) was contrasted with low invasivity (no use of CMM) as a reference. For analysis of deep CIPHEI, high invasivity (CMM with MER) was contrasted with medium/low invasivity (CMM without MER or no CMM at all) as a reference. The rationale for this differentiation was that extension of the sharp microelectrodes for MER comprises the most invasive aspect of the whole procedure (for deep CIPHEI), whereas the guiding cannula shields CMM from any parenchymatous contact >25 mm above the target.

The retrospective data analysis was originally planned to include a temporal cross-validation with two separate observation intervals, i.e., 2013–2019 and 2020–2021: the former larger time interval was envisaged for data exploration and establishing potential associations, whereas the latter shorter time interval was planned for cross-validation of findings.

**Figure 1:**
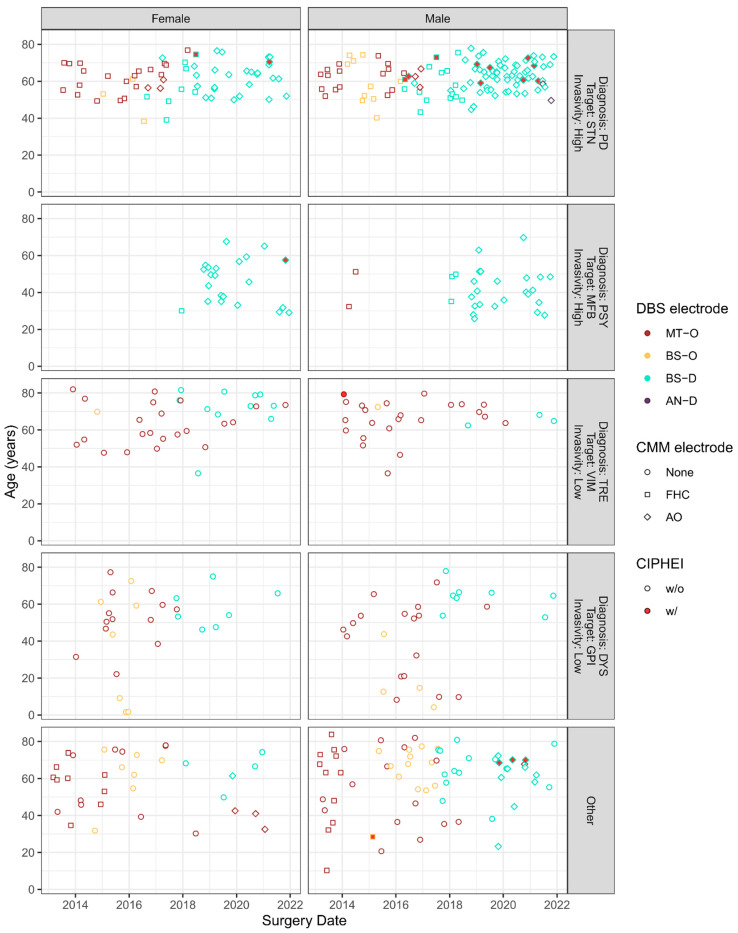
Across-time evolution of risk factor combinations for CIPHEI, such as target–diagnosis complexes, procedure-related variables (types of implant and combined micro/macroelectrodes), and patient-related variables (age and gender). Incidences of CIPHEI are highlighted by markers filled with red color.

However, inspection of Figure 1 on the distribution of factor combinations across time clearly indicated that factors were subject to substantial changes during the 9-year sampling interval—particularly with respect to procedure-related factors (e.g., transition from FHC (cf. Table 1 for implant abbreviations and specifications) to AO (cf. Table 1) of CMM around 2019, and transition from MT-O (cf. Table 1) and BS-O (cf. Table 1) omnidirectional DBS electrodes to BS-D (cf. Table 1) directional DBS electrodes around 2018), but also in combination with patient-related factors (e.g., adoption of high-invasive medial forebrain bundle (MFB)-DBS procedures in younger psychiatric patients around 2018, thereby almost exclusively using AO CMM in combination with BS-D DBS electrodes, and substantial reduction of low-invasive GPI- and VIM-DBS procedures in older dystonia and tremor patients around 2020, possibly reflecting the implications of COVID-19-associated shutdowns of elective treatments).

These time-associated changes introduced severe biases between the originally envisaged splits of the dataset, hence rendering a temporal cross-validation unfeasible. Considering that the vast majority of DBS procedures comprised bilateral implantations, we therefore decided to split the observations by hemisphere into an exploratory dataset (left-hemispheric procedures) and a cross-validation dataset (right-hemispheric procedures).

## 3. Results

### 3.1. Patient Sample

Characteristics of the overall sample are summarized in Supplementary Table S5. Retrospective sampling identified 436 consecutive procedures with 841 implanted electrodes (390 procedures with bilateral implantations, 89.4%; 31 procedures with unilateral implantations, 7.1%; 15 procedures with unilateral implantations of two electrodes, 3.4%) in 419 patients in total (170 females; median age, 60.7 years, interquartile range 50.1–68.2 years, range 1.6–83.8 years). One case with hemorrhage due to technical malfunction (defective auto-stop of the trepan) was excluded, resulting in 435 procedures (418 patients and 839 electrodes) submitted to further analyses (Supplementary Materials S2). Information on intraparenchymal (and extra-parenchymal) bleeding and factors of interest was available for all procedures.

### 3.2. CIPHEI Events

CIPHEI comprised a rare complication with 22 observed instances in 17 procedures only (5 procedures resulted in bilateral CIPHEI). The CIPHEI rates were 3.9% per procedure and 2.6% per electrode, which is within the range reported results [2,3,4,5,6,7,8,9,11,23,24,25,26,27,28,32,33,34,35,36,37,38,39]. Two procedures resulted in a permanent motor deficit (0.5%) and one in death of the patient (0.2%; see Supplementary Materials S2 for further descriptive and clinical information).

### 3.3. Confoundings between Factors

As evident from Supplementary Table S5, patient- and procedure-related factors were substantially confounded, as specific diagnoses entailed specific DBS approaches on specific targets in samples with specific sociodemographic and clinical characteristics. The sample comprised five major diagnosis–target–invasivity clusters (Figure 1), which were considerably confounded with age, gender, hypertension, and the types of CMM and DBS electrodes (Supplementary Materials S3). To at least partially resolve the confounding, we decided to omit diagnosis treated for and target for DBS as factors in the analyses.

### 3.4. Explorative Analysis (Left-Hemispheric Procedures)

Inspection of patient- and procedure-related characteristics in cases with left-hemispheric CIPHEI (Table 2) yielded several qualitative insights due to manifest accumulations of specific risk features: CIPHEI was observed (i) only in patients with an age ≥60 years, (ii) predominantly in male patients, (iii) only in medium- and high-invasive procedures (i.e., when using CMM/MER), but not in low-invasive procedures, and (iv) exclusively with BS-D implants. Furthermore, CIPHEI at any point of the trajectory and deep CIPHEI showed more frequent use of AO and FHC microelectrodes, respectively. There also appeared a prevalent target–diagnosis cluster of PD treated with subthalamic nucleus (STN)-DBS that was, however, almost perfectly confounded with the combination of high invasivity and older age and hence, not followed after. These descriptive observations were reflected in bivariable risk analyses (Supplementary Materials S6).

#### 3.4.1. Multivariable Analyses—Additive Combination of Factors

We analyzed full additive BR-GLM models, including main effects of all variables with subsequent backward elimination of (non-significant) terms. Age (≥60 years) and type of DBS electrode (BS-D vs. other) were binarized since CIPHEIs were exclusively observed in these instances (Table 2). For empirically resolving the inherent confounding between the use of CMM/MER (yes vs. no) and specific types of CMM (AO, FHC, and none), we entered both factors into the model, thus explicitly testing whether specific CMM types provided any additional predictive information.

For CIPHEI at any point of the trajectory, the final model revealed significantly increased odds ratios associated with age ≥60 years, use of CMM (irrespective of MER), and use of BS-D electrodes (Table 3). Albeit non-significant, male gender was kept as a factor, as its exclusion resulted in a poorer model fit in terms of a larger AIC (Supplementary Table S6-2). Specification of the CMM types resulted in a slightly better model fit than simply modeling the use of CMM (Supplementary Table S6-2), thus potentially indicating a higher CIPHEI risk when using FHC compared to AO CMM.

For deep CIPHEI, the final model included age ≥60 years, CMM with MER, and use of BS-D electrodes as independent factors (Table 3). Specification of CMM types again resulted in a far better model fit than the mere specification of microelectrode recording (Supplementary Table S6-3).

#### 3.4.2. Multivariable Analyses—Non-Additive Combination of Factors

For a meaningful evaluation of individual factors, an important question concerns whether the risk associated with the presence of a given factor is independent and stable or whether it is subject to variation dependent on the presence of other factors. From a statistical point of view, this refers to the concept of interaction (or moderation) between factors and their non-additivity.

The sparseness of CIPHEI, including complete and quasi-complete separation in the data, however, strictly limited the inclusion of interaction terms into the model. For instance, a full model with the 6 patient- and procedure-related factors would comprise a total of 57 predictors (including the intercept, the 6 simple effects, and 50 additional terms for 15 two-way, 20 three-way, 10 four-way, 4 five-way, and 1 six-way interaction), and is hence not statistically determinable since there were only 12 CIPHEI events in the left-hemispheric data (and 17 CIPHEI cases in the overall dataset). We, therefore, decided to reduce model complexity by focusing on a simple interaction model with a single non-additive combination of factors (i.e., the intercept and a single product term). This choice was also motivated by the qualitative observation in the data of a four-way quasi-complete separation by the accumulation of four risk features in left-hemispheric CIPHEI cases (Table 2). Model selection started with the six-way feature combination of all six patient- (age ≥60 years, male gender, and history of hypertension) and procedure-related factors (use of CMM/MER, CMM electrode type, and DBS electrode type) and continued by systematically eliminating factors from the product term based on the AIC.

For CIPHEI at any point of the trajectory, backward elimination (Supplementary Table S6-4) yielded a model with the non-additive combination of age ≥60 years, insertion of CMM (irrespective of MER), and implantation of BS-D electrodes (Table 3).

For deep CIPHEI, the final model comprised a four-way non-additive combination of age ≥60 years, CMM with MER, and use of FHC and BS-D product types (Table 3; Supplementary Table S6-5).

#### 3.4.3. Summary of Exploratory Risk Analyses in Left-Hemispheric Procedures

Additive and non-additive multivariable analyses consistently suggested the relevance of age ≥ 60 years, use of CMM/MER, and BS-D electrodes for significantly increasing CIPHEI risk. Comparing the applied (simple) additive and non-additive models based on AICs further suggested better model fits of the latter for explaining CIPHEI at any point of the trajectory and deep CIPHEI (Supplementary Materials S6). The explorative risk analyses thus indicated that CIPHEI risk was considerably elevated for multiplicative combinations of individual factors.

### 3.5. Cross-Validation Analysis (Right-Hemispheric Procedures)

Patient- and procedure-related characteristics of right-hemispheric CIPHEI cases are provided in Table 4 and resembled the insights from the left-hemispheric exploration data, namely, a preponderance of patients ≥ 60 years, male gender, medium/high-invasive procedures (CMM/MER), and BS-D electrodes. Bivariable analyses are reported in Supplementary Materials S7.

#### Multivariable Analyses—Additive and Non-Additive Combinations of Factors

Additive multivariable analyses with backward elimination did not converge in final models with significant terms (Supplementary Tables S7-2 and S7-3). For CIPHEI at any point of the trajectory, the final additive model included trends for age ≥60 years and use of CMM (irrespective of MER) as risk factors (Table 5). For deep CIPHEI, the final additive model comprised age ≥ 60 years and CMM with MER as risk factors and hypertension as a protective factor, with the former two at least marginally approaching a trend and the latter being non-significant (presumably acting as a suppressor variable; Table 5). Thus, cross-validation did not convincingly support the role of an additive combination of individual CIPHEI risk factors.

Non-additive multivariable analyses converged in the multiplicative combination of age ≥ 60 years, use of CMM/MER, and use of BS-D electrodes (Supplementary Tables S7-4 and S7-5), which significantly increased the odds ratios for both CIPHEI at any point of the trajectory and deep CIPHEI (Table 5). Model fits in terms of AICs again suggested the non-additive multiplicative feature combinations over the respective additive models (Supplementary Materials S7). The use of FHC CMM was not predictive in cases with deep CIPHEI.

### 3.6. Summary of Exploratory and Cross-Validation Analysis

**Figure 2:**
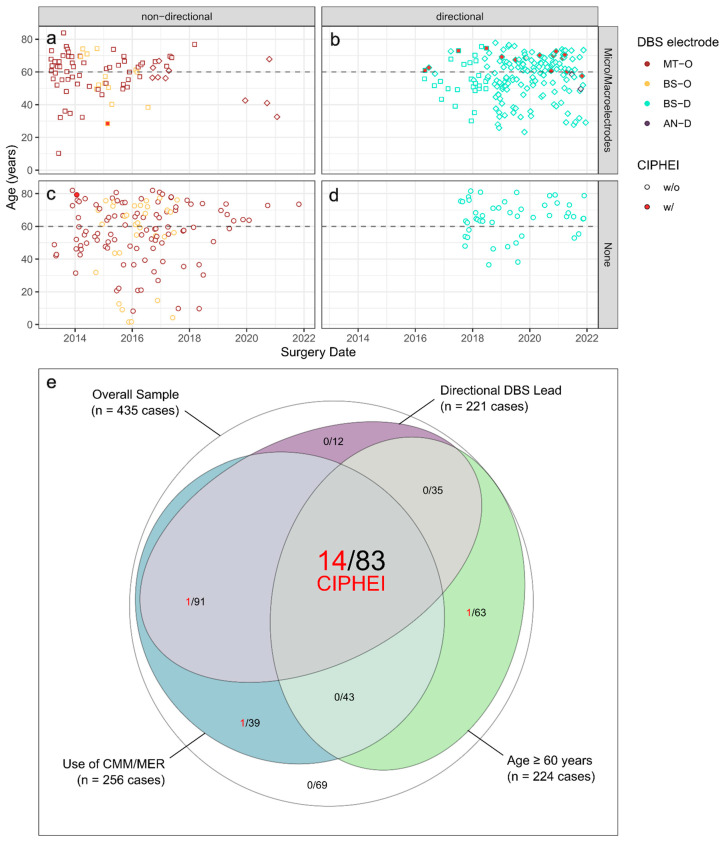
(**a**–**d**) Distribution of CIPHEI along factor combinations of older age (≥60 years, marked by dashed line), use of non-directional (**a**,**c**) vs. directional DBS electrodes (**b**,**d**), and medium to high level of invasivity (i.e., use of CMM/MER) (**b**,**d**) vs. low invasivity (no CMM/MER). CIPHEIs are clustered in (**b**) at ages older than 60 years. This dependency of CIPHEI on the three-way factor combination of older age (≥60 years), use of CMM/MER, and use of directional DBS electrodes is further highlighted in the (**e**) summary Euler diagram contrasting the counts of observed CIPHEIs (red font) with the respective basis counts of cases (black font) for all potential combinations.

Cross-validation provided clear-cut support for the non-additive combination of three factors associated with CIPHEI, which was identified in the exploratory analysis and comprised older age, medium/high-invasive procedure with CMM/MER, and use of BS-D electrodes. Figure 2 illustrates the non-additive interaction of these factors across the whole sample for all types of CIPHEI.

### 3.7. Additional Control Analyses

#### 3.7.1. Partial Dependency between the Exploration and Cross-Validation Samples

The cross-validation analyses have the limitation that the 10 underlying, right-hemispheric CIPHEI cases were not completely independent of the 12 left-hemispheric CIPHEI cases in the exploration sample considering bilateral CIPHEI in 5 patients. We, therefore, repeated the cross-validation of the final non-additive combination of factors focusing on the five patients with unilateral right-hemispheric CIPHEI at any point of the trajectory (Supplementary Table S8-1), whereas the three cases with deep CIPHEI were insufficient for repeating the respective cross-validation.

Results corroborated the relevance of the non-additive factor combination of older age, insertion of CMM (irrespective of MER), and use of BS-D electrodes for CIPHEI at any point of the trajectory (Supplementary Materials S8).

#### 3.7.2. Coagulation and Platelet Function in CIPHEI Patients

For 10 out of 17 CIPHEI cases, a full postoperative coagulation work-up was available but not indicative of a relevant role of coagulation or platelet function, as only one CIPHEI patient showed acquired platelet dysfunction (Supplementary Materials S4).

## 4. Discussion

The retrospective analysis of a large, consecutive, single-center series of DBS implantations provided two new insights into CIPHEI risk factors: First, both exploration and cross-validation analysis suggested directional implants as a potential CIPHEI risk factor, whereas older age and medium/high invasivity (i.e., CMM/MER) were confirmed as further risk factors. Second, model comparisons clearly favored a non-additive/multiplicative combination of these three factors, thus suggesting that CIPHEI risk was not driven by the individual factors alone but by their combined presence.

As a new finding, we encountered at our center that the choice of DBS implant is of relevance, with a higher CIPHEI frequency in patients receiving directional BS-D implants. However, Figure 2 impressively illustrated that directional BS-D implants (cyan markers) could be safely used when non-invasively implanted (Figure 2d) or even with CMM/MER when implanted in younger patients (Figure 2b). Only the combination of older age, CMM/MER, and directional BS-D implants substantially boosted CIPHEI risk in our sample (Figure 2e).

Notably, this mutual three-way interaction accounted for 82.4% of the CIPHEI cases (14 out of 17), with one-third even exhibiting bilateral events (5 of 14 CIPHEI cases with the three-way risk combination; Supplementary Materials S9), while the overall CIPHEI frequency was in line with previous results [2,3,6,7,23].

But what drives this interaction of risk factors? Considering that methodology (i.e., invasivity and implanted electrode) and even trajectories for STN-DBS and medial forebrain bundle-DBS closely resemble each other, whereas the respective CIPHEI frequencies substantially differ, a (hidden) confounding with another procedure-related factor is unlikely. Likewise, the interaction’s persistence across different diagnoses mitigates possible effects of (hidden) patient-related confounding.

A potential but speculative explanation may concern a mutual reaction between idiosyncrasies of brain parenchyma in the elderly once transgressed by CMM and the coating and/or the design of the directional electrode.

Older age has previously been associated with intracranial hemorrhage [3,8,9,10,11,12], with age-related atrophy and vascular stiffness potentially increasing vulnerability [40].

Invasivity in terms of CMM/MER may drive CIPHEI via two distinct mechanisms: As CIPHEI at any point of the trajectory (among them superficial ones >25 mm above target) occurred with CMM irrespective of MER, the guiding cannulas used for CMM insertion obviously pose a relevant risk. In turn, deep CIPHEI occurred exclusively with CMM and extended microelectrodes for MER, suggesting the sharp microelectrodes as a potential cause. These associations are implicitly supported by a study without employment of CMM/MER, reporting lower hemorrhage rates [3], but higher rates also exist [8].

The role of directional electrodes in promoting CIPHEI has not been described before. Compensating for small inaccuracies in electrode placement, directional electrodes can expand the therapeutic window of stimulation [41]. We used guiding cannulas for inserting CMM but not for DBS electrodes, which is a more common procedure in Europe compared with the USA. Microscopic sharpness of DBS electrode contacts could hence further exaggerate the vulnerability introduced by the other two factors: older age and use of CMM. Alternatively, agents in the coating of directional electrodes could interfere with coagulation/platelet aggregation. In fact, several recent conference papers showed that switching from omnidirectional to directional electrodes (regardless of manufacturer) led to increased incidences of peri-lead edema across different DBS centers [42,43,44,45]. Despite being a different entity than CIPHEI, this rise in peri-lead edema indicates that directional DBS electrodes may interact differently with brain tissue than omnidirectional DBS electrodes. Usage of a guiding cannula for DBS electrode placement might hence reduce CIPHEI by minimizing tissue contact during insertion.

Surprisingly, we observed only one CIPHEI with Medtronic’s non-directional 3389/3387 electrodes, whereas other authors have repeatedly reported such cases [3,8,10,16,17,28,32,46,47,48], matching our experience during past employment at another hospital.

Male gender was not convincingly supported as a risk factor, possibly reflecting a power issue (Supplementary Materials S6 and S7). As male gender has been infrequently identified as a risk factor [12,13], it might become relevant only in specific risk factor combinations.

A history of arterial hypertension was not identified as a risk factor here but has been reported by some [3,8,9,15], possibly reflecting increased vulnerability due to elevated vascular stiffness. However, intraoperative blood pressure might be a more comprehensible CIPHEI risk factor, which is not necessarily tied to a history of arterial hypertension [13,14].

The relevance of CMM type for deep CIPHEI remained inconclusive regarding FHC as a risk factor (Supplementary Materials S6 and S7), although this association has been reported in the past [10].

Regarding complications of DBS surgery, the surgical learning curve has been discussed [24,25,33]. Of the three surgeons involved, one has six and two over twenty years of experience in DBS surgery. Although we could not differentiate individual contributions (as implantations were usually alternated between two surgeons, with one in the lead), CIPHEIs appeared distributed according to the surgeons’ shares of lead responsibility. The increasing CIPHEI frequency across time (Figure 2) further contradicts a learning-curve hypothesis.

Several limitations apply to our study. Delayed asymptomatic CIPHEIs may have been missed, as postoperative imaging was not routinely performed after the day of surgery, yet this applies to most studies in the field.

Intraoperative blood pressure values were monitored and manually documented but not digitally recorded. However, standards of care regarding intraoperative blood pressure monitoring and treatment remained unchanged over the observational period and cannot explain the increase in CIPHEIs.

This study used retrospective data from a single center and CIPHEI is a rare complication of DBS implantation, which limits generalizability and impedes disentangling confounded risk factors. Although we applied multivariable analysis with bias reduction for sparse sampling, we cannot rule out that potential associations remained undetected due to insufficient power (which may possibly apply to the unclear role of male gender). Procedural changes did not occur to the implantation of DBS electrodes in the analyzed time period, apart from the change in the mainly used CMM and DBS electrodes (see Figure 1).

Exploration and cross-validation samples were not completely independent considering the bilateral CIPHEIs in some patients. Results were corroborated by control analyses in non-overlapping patients, whereas the non-random occurrence of bilateral CIPHEI further underlines the relevance of risk factor combinations (Supplementary Materials S8 and S9).

Preoperative platelet functioning, which can be an issue for intracranial hemorrhage in DBS surgery [49], was not monitored. However, available postoperative data on coagulation and platelets in 10 out of 17 CIPHEI patients did not indicate a relevant contribution of platelet dysfunction.

## 5. Conclusions

CIPHEI risk is driven by a mutual interaction of older age, medium/high invasivity (CMM/MER), and directional DBS electrodes. Considering that superficial CIPHEIs did not have contact with the actual CMM, the mere insertion of the guiding cannulas seemed to pose a relevant risk in this interaction. Modifiable procedure-related choices should be individually adjusted, whereas patients requiring DBS treatment at some point in time should be considered in early disease stages. Further prospective, preferably multi-center studies are warranted to investigate the role of specific implants as CIPHEI risk factors.

## Figures and Tables

**Table 1:** Overview of the applied types of CMM and DBS electrodes.

Electrode Type	Producer	Product Type and Name	Abbr.
CMM electrode	Alpha-Omega (Nof HaGalil, Israel)	Sonos Shielded Microelectrode, model STR-009080-00	AO
	FHC (Bowdoin, ME, USA)	microTargeting D.ZAP Array Insertion Electrode, model FC2001	FHC
DBS electrode	Medtronic (Minneapolis, MN, USA)	quadripolar omnidirectional models 3389 or 3387	MT-O
	Boston Scientific Corp. (Marlborough, MA, USA)	octopolar omnidirectional model 2201	BS-O
		octopolar directional model 2202	BS-D
	Aleva Neurotherapeutics (Lausanne, Switzerland)	dodecapolar directional model 11500	AO

Note. The abbreviations in the right-most column are used in the main text as references to the specific electrode types. The two types of combined micro/macroelectrodes (CMM) are referred to by acronyms of the producer’s name only, whereas the four types of deep brain stimulation (DBS) electrodes are further differentiated by their directionality (i.e., O, omnidirectional; D, directional).

**Table 2:** Characteristics of cases with left-hemispheric CIPHEI.

CIPHEI					Procedure-Related Variables			Patient-Related Variables		
≤25 mm	>25 mm	Invasivity	Diagnosis	Target	CMM Electrode	DBS Electrode	Laterality	Age (Years)	Gender	aHTN
	x	medium	PAI	VCP + PAG	AO	BS-D	Left	68.6	M	w/o
	x	medium	TRE	VIM	AO	BS-D	Bilateral	70.2	M	w/o
	x	medium	TRE	VIM	AO	BS-D	Bilateral	70.1	M	w/
	x	high	PD	STN	AO	BS-D	Bilateral	69.2	M	w/o
	x	high	PD	STN	AO	BS-D	Bilateral	67.4	M	w/o
	x	high	PD	STN	AO	BS-D	Bilateral	60.7	M	w/
	x	high	PD	STN	AO	BS-D	Left	72.6	M	w/
	x	high	PD	STN	AO	BS-D	Bilateral	68.4	M	w/
x		high	PD	STN	FHC	BS-D	Bilateral	74.5	F	w/o
x	x	high	PD	STN	FHC	BS-D	Bilateral	60.9	M	w/o
x	x	high	PD	STN	FHC	BS-D	Left	73.0	M	w/
x	x	high	PD	STN	AO	BS-D	Bilateral	60.2	M	w/o

Abbreviations: CIPHEI, cerebral intraparenchymal hemorrhage due to electrode implantation. DBS, deep brain stimulation. CMM, combined micro/macroelectrodes. Please refer to Table 1 for the specifications of CMM and DBS electrode types. Diagnoses: PAI, chronic pain; PD, Parkinson’s disease; TRE, non-Parkinsonian tremor. Targets: PAG, periaqueductal gray; STN, subthalamic nucleus; VIM, ventral intermediate nucleus of the thalamus; VCP, parvocellular part of ventrocaudal nucleus of the thalamus. Laterality refers to laterality of implantation: unilateral implantations in STN resulted from termination of surgery due to CIPHEI with intraoperative symptom onset. Gender: M, male; F, female. aHTN, arterial hypertension: w/, with; w/o, without.

**Table 3:** Model estimates for left-hemispheric CIPHEI (exploration analysis).

		Model Estimates					
Model Type	Dependent Variable	Term	Log OR	SE	Statistic	*p*-Value	OR [95% CI]
Additive	Any CIPHEI along trajectory	Intercept	−12.154	2.559	−4.749	**0.000002**	0.00001 [<0.00001–0.00041]
		Age (≥60 years)	3.587	1.383	2.593	**0.009511**	36.12 [4.36–Inf]
		Gender (Male)	1.310	0.856	1.531	0.125791	3.71 [0.79–106.73]
		CMM electrode type (FHC)	3.473	1.518	2.287	**0.022189**	32.23 [2.60–Inf]
		CMM electrode type (AO)	2.098	1.414	1.484	0.137831	8.15 [0.97–Inf]
		DBS electrode type (BS-D)	3.524	1.480	2.381	**0.017269**	33.94 [2.85–Inf]
	Deep CIPHEI	Intercept	−10.772	2.561	−4.206	**0.000026**	0.00002 [<0.00001–0.00167]
		Age (≥60 years)	3.017	1.477	2.043	**0.041048**	20.43 [1.83–Inf]
		CMM electrode type (FHC)	3.504	1.534	2.285	**0.022317**	33.25 [2.72–Inf]
		CMM electrode type (AO)	0.303	1.611	0.188	0.850981	1.35 [0.07–Inf]
		DBS electrode type (BS-D)	3.591	1.504	2.388	**0.016942**	36.27742 [3.01–Inf]
Non-additive	Any CIPHEI along trajectory	Intercept	−6.509	1.417	−4.592	**0.000004**	0.00149 [<0.00001–0.01003]
		Age (≥60 years) × use of CMM/MER × DBS electrode type (BS-D)	4.793	1.451	3.304	**0.000953**	120.68 [15.66–Inf]
	Deep CIPHEI	Intercept	−5.602	0.819	−6.840	**<0.000001**	0.00369 [0.00014–0.01313]
		Age (≥60 years) × use of CMM/MER × DBS electrode type (BS-D)	4.983	1.077	4.628	**0.000004**	145.92 [20.93–4458.38]

Abbreviations: CIPHEI, cerebral intraparenchymal hemorrhage due to electrode implantation. DBS, deep brain stimulation. CMM, combined micro/macroelectrodes; MER, microelectrode recording; SE, standard error; OR, odds ratio; CI, confidence interval. Due to sparse sampling of CIPHEI, for some terms, the upper bound of the OR’s confidence interval was only estimable with large uncertainty (Inf, infinity). The estimate for the intercept reflects the odds for a CIPHEI, whereas the estimates for the terms reflect their respective ORs. *p*-values < 0.05 are marked in bold font.

**Table 4:** Characteristics of cases with right-hemispheric CIPHEI.

CIPHEI					Procedure-Related Variables			Patient-Related Variables		
≤25 mm	>25 mm	Diagnosis	Target	Invasivity	CMM Electrode	DBS Electrode	Laterality	Age (Years)	Gender	aHTN
	x	TRE	VIM	Low	None	MT-O	Bilateral	79.3	M	w/
	x	TRE	VIM	Medium	AO	BS-D	Bilateral	70.1	M	w/
	x	PD	STN	High	AO	BS-D	Bilateral	69.2	M	w/o
	x	PD	STN	High	AO	BS-D	Bilateral	68.4	M	w/
	x	PSY	MFB	High	AO	BS-D	Bilateral	57.5	F	w/o
x		DYS	GPI	High	FHC	BS-O	Bilateral	28.4	M	w/o
x		PD	STN	High	FHC	BS-D	Bilateral	60.9	M	w/o
x		PD	STN	High	AO	BS-D	Bilateral	62.7	M	w/o
x		PD	STN	High	AO	BS-D	Right	70.5	F	w/o
x	x	PD	STN	High	AO	BS-D	Bilateral	67.4	M	w/o

Abbreviations: CIPHEI, cerebral intraparenchymal hemorrhage due to electrode implantation. DBS, deep brain stimulation. CMM, combined micro/macroelectrodes. Please refer to Table 1 for the specifications of CMM and DBS electrode types. Diagnoses: DYS, dystonia; PD, Parkinson’s disease; PSY, psychiatric disease (i.e., therapy-refractory depression or obsessive-compulsive disorder); TRE, non-Parkinsonian tremor. Targets: GPI, globus pallidus internus; MFB, medial forebrain bundle; STN, subthalamic nucleus; VIM, ventral intermediate nucleus of the thalamus. Laterality refers to laterality of implantation: unilateral implantations in STN resulted from termination of surgery due to CIPHEI with intraoperative symptom onset. Gender: M, male; F, female. aHTN, arterial hypertension: w/, with; w/o, without.

**Table 5:** Model estimates for right-hemispheric CIPHEI (cross-validation analysis).

		Model Estimates					
Model Type	Dependent Variable	Term	Log OR	SE	Statistic	*p*-Value	OR [95% CI]
Additive	Any CIPHEI along trajectory	Intercept	−5.505	0.992	−5.548	**<0.000001**	0.00407 [0.00010–0.02121]
		Age (≥60 years)	1.296	0.717	1.808	0.070609	3.66 [0.99–28.90]
		Use of CMM	1.503	0.873	1.722	0.085038	4.50 [1.02–118.78]
	Deep CIPHEI	Intercept	−6.188	1.465	−4.223	**0.000024**	0.00205 [0.00000–0.02052]
		Age (≥60 years)	1.447	0.902	1.604	0.108697	4.25 [0.77–112.87]
		aHTN (w/)	−1.660	1.379	−1.204	0.228537	0.19 [<0.00001–1.76]
		Use of CMM/MER	1.996	1.349	1.479	0.139059	7.36 [0.82–Inf]
Non-additive	Any CIPHEI along trajectory	Intercept	−4.536	0.538	−8.428	**<0.000001**	0.01072 [0.00228–0.02637]
		Age (≥60 years) × use of CMM/MER × DBS electrode type (BS-D)	2.267	0.662	3.423	**0.000620**	9.65 [2.80–49.92]
	Deep CIPHEI	Intercept	−5.404	0.820	−6.594	**<0.000001**	0.00450 [0.00019–0.01472]
		Age (≥60 years) × use of CMM/MER × DBS electrode type (BS-D)	2.653	0.955	2.779	**0.005456**	14.19 [1.70–229.18]

Abbreviations: CIPHEI, cerebral intraparenchymal hemorrhage due to electrode implantation. DBS, deep brain stimulation. CMM, combined micro/macroelectrodes. MER, microelectrode recording. SE, standard error. OR, odds ratio. CI, confidence interval. Due to sparse sampling of CIPHEI, for some terms, the upper bound of the OR’s confidence interval was only estimable with large uncertainty (Inf, infinity). The estimate for the intercept reflects the odds for a CIPHEI, whereas the estimates for the terms reflect their respective ORs. *p*-values < 0.05 are marked in bold font.

## Data Availability

Data will be made available upon reasonable request and after approval of the institutional review board.

## Supplementary Materials

The following supporting information can be downloaded at: https://www.mdpi.com/article/10.3390/brainsci14060612/s1. The supplementary file contains the following sections: Abbreviations, S1. DBS Electrode Implantation, S2. Hemorrhagic Events, S3. Confounding between Risk Factors for CIPHEI, S4. Coagulation and Platelet Function, S5. Characteristics of the Overall Sample, S6. Explorative Analysis (Left-Hemispheric Procedures), S7. Cross-Validation Analysis (Right-Hemispheric Procedures), S8. Additional Control Analyses, S9. Further Considerations, and References for Supplementary Materials.

## Abbreviations

AIC	Akaike Information Criterion
AN-D	Aleva Neurotherapeutics dodecapolar directional DBS electrode model 11500
AO	Alpha Omega Sonos Shielded Microelectrode
BR-GLM	Bias reduction in binomial-response generalized linear models
BS-D	Boston-Scientific octopolar directional DBS electrode model 2202
BS-O	Boston-Scientific octopolar omnidirectional DBS electrode model 2201
CIPHEI	cerebral intraparenchymal hemorrhage due to electrode implantation
CMM	combined micro/macroelectrodes
CT	computed tomography
DBS	deep brain stimulation
FHC	FHC microtargeting electrode
MER	microelectrode recording, i.e., extending microelectrodes of combined micro/macroelectrodes
MFB	medial forebrain bundle
MT-O	Medtronic quadripolar omnidirectional DBS electrode models 3389 or 3387
PD	Parkinson’s disease
STN	subthalamic nucleus
