# Supplementary material for: Cerebral Intraparenchymal Hemorrhage due to Implantation of Electrodes for Deep Brain Stimulation: Insights from a Large Single-Center Retrospective Cross-Sectional Analysis
Source: Brain Sci. 2024 Jun 19;14(6):612. doi: 10.3390/brainsci14060612 (PMC11201406; doi:10.3390/brainsci14060612)
Supplement: Supplementary file 1 [file brainsci-14-00612-s001.zip › brainsci-3011774-supplementary.pdf]

Incidence and Interaction of Predictors of Cerebral Intraparenchymal Hemorrhage due to Implantation of Electrodes for Deep Brain Stimulation: a Large Single-Center Retrospective Cross-Sectional Study

Bastian E. A. Sajonz, Timo S. Brugger, Marco Reisert, Martin Büchsel, Nils Schröter, Alexander Rau, Karl Egger, Peter C. Reinacher, Horst Urbach, Volker A. Coenen, Christoph P. Kaller

SUPPLEMENTARY MATERIALS

Table of Contents

Abbreviations 2

S1 DBS Electrode Implantation 3

S2 Hemorrhagic Events 5

S3 Confoundings between Risk Factors for CIPHEI 6

S4 Coagulation and Platelet Function 10

S5 Characteristics of the Overall Sample 11

S6 Explorative Analysis (Left-Hemispheric Procedures) 13

S7 Cross-Validation Analysis (Right-Hemispheric Procedures) 19

S8 Additional Control Analyses 24

S9 Further Considerations 26

References of Supplementary Material 27

Abbreviations

AIC	Akaike information criterion
AO	Alpha Omega Sonos Shielded Microelectrode
BS-D	Boston-Scientific octopolar directional DBS electrode model 2202
BS-O	Boston-Scientific octopolar omnidirectional DBS electrode model 2201
CIPHEI	cerebral intraparenchymal hemorrhage due to electrode implantation
CMM	combined micro/macro-electrodes
Col/ADP	collagen/adenosine diphosphate
Col/Epi	collagen/epinephrin
CT	computed tomography
DBS	deep brain stimulation
FDG	[^{18}F]fluorodeoxyglucose
FHC	FHC microtargeting electrode
GPI	globus pallidus internus
LTA	light transmission aggregometry
MER	microelectrode recording, i.e. extending microelectrodes of combined micro/macroelectrodes
MFB	medial forebrain bundle
MRI	magnetic resonance imaging
MT-O	Medtronic quadripolar omnidirectional DBS electrode models 3389 or 3387
NSAID	non-steroidal anti-inflammatory drug
PET	positron emission tomography
PFA	platelet function analyzer
STN	subthalamic nucleus
VIM	nucleus ventralis intermedius thalami
VWF	von Willebrand factor

S1 DBS Electrode Implantation

If part of the patient’s medication, anticoagulants, or thrombocyte aggregation inhibitors were paused prior to admission according to institutional standards. All patients had normal preoperative partial thromboplastin time, prothrombin time and no relevant thrombocytopenia. Preliminary trajectories were planned based on isotropic high resolution T2- and contrast enhanced T1-weighted magnetic resonance imaging (MRI) and combined with fiber tracking based on diffusion tensor imaging if required. In cases of a two-staged implantation of DBS electrodes the same MR images were used for stereotactic planning of both operations. The DBS implantation was based solely on computed tomography (CT) imaging in four patients (n=3 GPI, n=1 VIM) with functional implants (n=2 cochlear implants, n=2 implantable cardioverter-defibrillator) impeding MRI acquisition due to safety or imaging quality issues.
For stereotactic surgery, a Leksell G-frame (Elekta, Sweden) was mounted on the patient's head. A CT scan was performed with a localizer box (Elekta, Sweden). Contrast agent was applied for CT-angiography whenever feasible. After fusion of the CT(-angiography) and MRI data, the trajectories were adjusted to avoid vessels and sulci. A 14 mm burr hole was placed at the entry point of the trajectory and waxed (with bone wax, B.Braun, Melsungen, Germany). to avoid osseous or epidural bleeding. A burr hole cover was fixed at the burr hole. Bipolar coagulation was performed prior to incision at the dural entry point followed by bipolar corticotomy. Standard of care includes blood pressure monitoring with an upper arm cuff every 5 minutes and treatment to keep systolic pressure below 160 mmHg.
In case of planned MER guiding cannulas corresponding to the number of planned MER-paths (usually 2-3) were lowered and the dural defect was sealed with fibrin glue (Tisseel, Baxter, Deerfield, IL, USA). CMMs (Sonos Shielded Microelectrode, model STR-009080-00 from Alpha-Omega, Nof HaGalil, Israel; or microTargeting D.ZAP Array Insertion Electrode, model FC2001 from FHC, Bowdoin, ME, USA) were then introduced via the guiding cannulas and lowered with a microdrive into the target region with extended microelectrodes. MER was started 10 mm above target and repeated in 0.5 mm steps until reaching the target point and only extended up to 2 mm below target for verification of the lower border if necessary.
Guiding cannulas and CMM were always aligned in a plus (+) configuration of the Ben-Gun array with outer parallel tracks being offset by 2 mm to the center track. Corresponding to the CMM model we used a proprietary microdrive (Neuro Omega Autoclavable Drive Headstage, model 750-000025-00, Alpha-Omega, Nof HaGalil, Israel; or STar Drive Manual, model ST-DS-MA, FHC, Bowdoin, ME, USA) and proprietary guiding cannulas (Stainless Steel Guide Tube and Stylet, outer diameter 1.8 mm, model STR-000021-00, Alpha-Omega, Nof HaGalil, Israel; or microTargeting STar Insertion Tube, outer diameter 0.9 mm, model FC8009, FHC, Bowdoin, ME, USA).
The Alpha-Omega guiding cannulas end 25 mm above target, while the FHC Insertion Tube ends 20 mm above target. As all deep CIPHEIs with FHC CMM originated within 20 mm above target, this difference in length did not affect our classification into deep vs. superficial CIPHEIs. To simplify and improve readability throughout tables and the article, we set 25 mm above target (irrespective of the actually applied guiding cannula) as the limit to differentiate deep from superficial CIPHEIs.
In surgeries without MER, either a single blunt-tip monopolar lesion electrode (Cosman, Burlington, MA, USA, or Inomed, Emmendingen, Germany) was advanced with the above mentioned microdrive from FHC (Bowdoin, ME, USA) or, in a few cases, two CMMs with retracted microelectrodes were used instead with their corresponding proprietary microdrive

and guiding cannula as described above. In those cases microelectrodes were retracted so they do not protrude above the tip of the macroelectrode.

Target verification methods included electrophysiological and clinical examination and were applied according to target-point specific institutional standards: e.g. MER was typically applied for the targets STN and MFB but rarely for GPI and never for VIM. Finally, testing electrodes (CMMs or blunt-tip electrodes) were moved to the desired implantation depth verified by intraoperative lateral X-ray.

After retraction of the microelectrode and guiding cannula or blunt-tip electrode from the desired track a DBS electrode (quadripolar omnidirectional models 3389 or 3387 from Medtronic, Minneapolis, MN, USA; octopolar omnidirectional model 2201 or octopolar directional model 2202 (Vercise Cartesia) from Boston-Scientific, Marlborough, MA, USA; dodecapolar model 11500 (directSTIM) from Aleva Neurotherapeutics, Lausanne, Switzerland) was lowered to the target region and anchored in the burr hole cover after fluoroscopic verification. For initially bilateral procedures the order of implantation was determined by the patients' wish for stimulator placement with the contralateral electrode being implanted first and the ipsilateral DBS electrode second.

Postoperative helical CT was conducted on the day of surgery and assessed to corroborate the final electrode position and to screen for signs of hemorrhage. Additional CT scans were obtained in case of delayed onset of new neurological symptoms after the day of surgery.

S2 Hemorrhagic Events

Descriptives

Intracranial hemorrhagic events comprised a rare complication that was observed in 25 procedures only, of which 9 and 18 bleedings were considered of extra- and intraparenchymal origin, respectively, with an overlap in 2 procedures that each showed two spatially clearly distinct extra- and intraparenchymal hemorrhages.

The resulting overall intracranial hemorrhage (i.e. combined extra- and intraparenchymal) rates comprised 5.7% per procedure and 3.7% per electrode. Furthermore, one case with bilateral superficial CIPHEI due to technical malfunction (defective autostop of the trepan) was excluded in the subsequent analyses, as the contusional bleeding would have occurred in any patient regardless of the analyzed risk factors. The CIPHEI rates in the resulting 17 valid cases with CIPHEI were 3.9% per procedure and 2.6% per electrode.

Although intracerebral hemorrhage due to DBS implantation is the second most common procedure-related complication after perioperative mental status change [1,2], it is overall rare. The present rate of 3.9% per procedure and 2.6% per electrode is within the range of previously reported results [1–23].

However, direct comparisons require caution, as e.g. the differentiation between intraparenchymal/extraparenchymal hemorrhages is often not available or clearly indicated [1,7,13–16,18–20].

Neurological Symptoms

Six out of seven patients with exclusively extraparenchymal hemorrhage did not show any associated symptoms, whereas one patient displayed dysphasia that completely resolved until discharge.

Five out of the included 17 patients with CIPHEI showed immediate neurological symptoms during surgery, while another 9 developed symptoms in the postoperative course on the ward. The remaining three patients showed no symptoms at any time during the treatment. Symptoms resolved in all but three patients (one patient died due to CIPHEI and two had a residual hemiparesis). Despite the intraoperative onset of new neurological symptoms, the CT scan showed no signs of hemorrhage in one patient, but the CIPHEI was verified in a subsequent MR scan. Four patients had a delayed CIPHEI, which was not apparent on the postoperative CT scan but on additional CT scans obtained due to delayed onset (2-6 days) of symptoms.

S3 Confoundings between Risk Factors for CIPHEI

Prevalence of Diagnosis-Target-Invasivity Clusters

The overview of patient- and procedure-related characteristics (Supplementary Table S5) revealed that 337 of the 436 procedures (77.3%) were covered by only four of the 22 overall observed combinations of diagnosis, target, and invasivity: (i) high-invasive STN-DBS in patients with Parkinson’s disease (n = 170, 39.0%) (ii) high-invasive MFB-DBS in psychiatric patients (n = 50, 11.5%) (iii) low-invasive VIM-DBS in tremor patients (n = 61, 14.0%), and (iv) low-invasive GPI-DBS in dystonia patients (n = 56, 12.8%).

Confoundings between the resulting five diagnosis-target-invasivity clusters (i.e., the four aforementioned plus the remaining patients) were analyzed using a general linear model for continuous age and log-linear models on the frequency distributions for the remaining discrete risk factors. Omnibus tests (type III sums of square) are reported in Supplementary Table S3-1 revealing substantial differences between the different diagnosis-target-invasivity clusters with respect to the distributions of age (Supplementary Figure S3-1a), gender (Figure S3-1b), hypertension (Figure S3-1c), and the utilized types of micro/ macroelectrodes (Figure S3-1d, restricted to medium- to high-invasive procedures), and types of DBS electrodes (Figure S3-1e).

Table S3-1. Confoundings between diagnosis-target-invasivity clusters and other risk factors.

Independent variable	Dependent variable	Statistic	df	p-value
Diagnosis-Target-Invasivity Cluster	Age	132.99	4	<0.000001
	Gender	10.29	4	0.035840
	aHTN	18.54	4	0.000967
	CMM electrode	23.76	4	0.000089
	DBS electrode	138.58	12	<0.000001

Note. Omnibus tests were based on likelihood-ratio chi square test statistics. Abbreviations: aHTN, history of arterial hypertension; CMM, combined micro/macro-electrode; DBS, deep brain stimulation.

Due to these considerable confoundings of diagnosis-target-invasivity clusters with other risk factors, we decided to omit the diagnosis treated with and the target for DBS as risk factors.

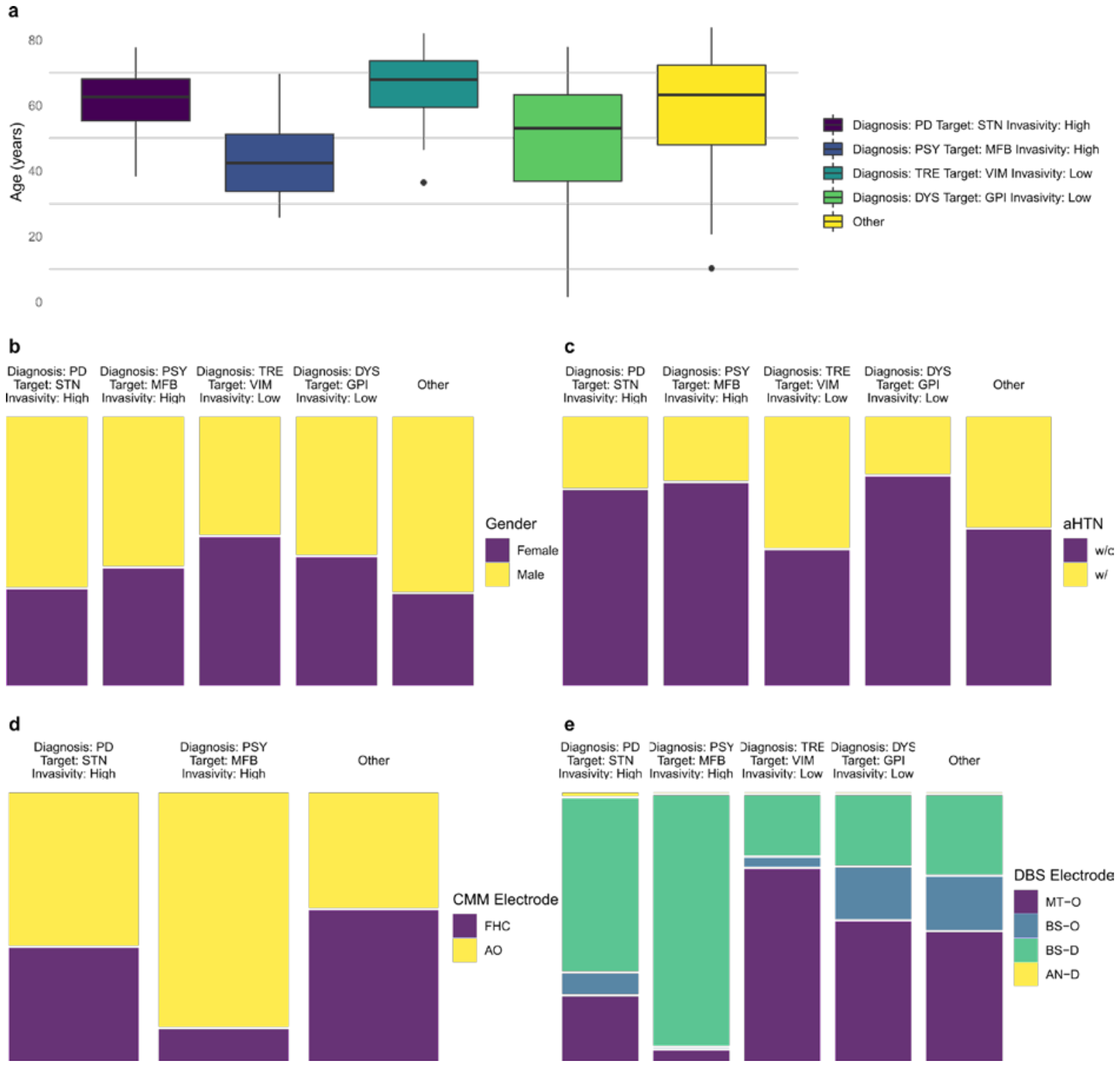

Figure S3-1. Distributions of (a) age, (b) gender, (c) arterial hypertension, (d) type of CMM electrode (limited to medium- and high-invasive procedures, otherwise trivial), and (e) type of DBS electrode type as a function of diagnosis-target-invasivity clusters. Abbreviations: CMM, combined micro/macro-electrodes. Diagnoses: DYS, dystonia; PD, Parkinson's disease; PSY, psychiatric disease (i.e. therapy-refractory depression or obsessive-compulsive disorder); TRE, non-Parkinsonian Tremor. Targets: GPI, globus pallidus internus; MFB, medial forebrain bundle; STN, subthalamic nucleus; VIM, ventral intermediate nucleus of the thalamus. aHTN, arterial hypertension: w/, with; w/o, without. Please refer to Table 1 (main text) for the specification of CMM and DBS electrode types.

Confoundings between Patient- and Procedure-Related Risk Factors

Confoundings between the remaining patient- and procedure-related risk factors of interest were explored using pair-plots (Supplementary Figure S3-2) followed-up by inferential statistics. To this end, we analyzed separate general linear models for the different discrete risk factors on continuous age, whereas confoundings between the different bivariable combinations of discrete risk factors were addressed with log-linear models on the frequency distributions assuming Poisson distributions (see Supplementary Table S3-2 for results of omnibus tests, type III sums of square).

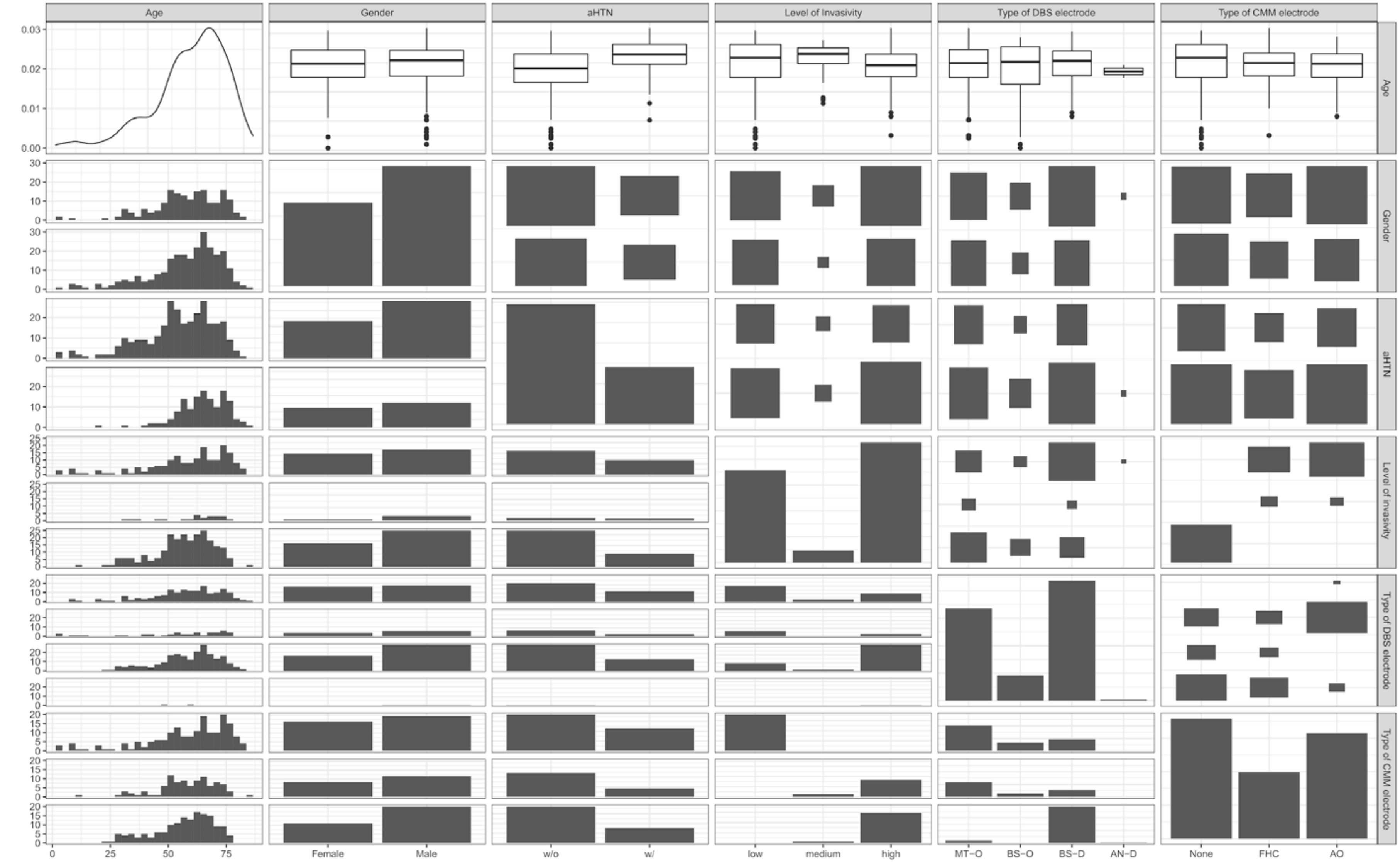

Figure S3-2. Pair plot for inspecting potential confoundings between the risk factors of interest. Abbreviations: aHTN, history of arterial hypertension; CMM, combined micro/macro-electrode; DBS, deep brain stimulation. Please refer to Table 1 for the specification of CMM and DBS electrode types.

Table S3-2. Confoundings between other risk factors.

Independent variable	Dependent variable	Statistic	df	p-value
Gender	Age	<0.01	1	0.992178
	aHTN	0.96	1	0.326196
	Level of invasivity	5.89	2	0.052729
	Type of DBS electrode	7.01	3	0.071546
	Type of CMM electrode	4.05	2	0.131779
aHTN	Age	50.29	1	<0.000001
	Level of invasivity	7.40	2	0.024752
	Type of DBS electrode	3.91	3	0.271780
	Type of CMM electrode	4.94	2	0.084406
Level of invasivity	Age	2.34	2	0.309786
	Type of DBS electrode	96.52	6	<0.000001
	Type of CMM electrode	594.42	4	<0.000001
Type of DBS electrode	Age	3.56	3	0.313403
	Type of CMM electrode	213.61	6	<0.000001
Type of CMM electrode	Age	0.91	2	0.634586

Note. Omnibus tests were based on likelihood-ratio chi square test statistics. Abbreviations: aHTN, arterial hypertension; CMM, combined micro/macro-electrode; DBS, deep brain stimulation.

Results revealed several significant associations and trends thereof: (i) gender and level of invasivity ($p = 0.053$), (ii) gender and type of DBS electrode ($p = 0.072$), (iii) history of arterial hypertension and age ($p < 0.001$), (iv) history of arterial hypertension and level of invasivity ($p = 0.025$), (v) history of arterial hypertension and type of CMM electrode ($p = 0.084$), (vi) level of invasivity and type of DBS electrode ($p < 0.001$), (vii) level of invasivity and type of CMM electrode ($p < 0.001$), and (viii) type of DBS electrode and type of CMM electrode ($p < 0.001$).

S4 Coagulation and Platelet Function

Postoperative values of coagulation and platelet function of patients with CIPHEI were assessed in a separate exploratory analysis. We measured activated Partial Thromboplastin Time (Pathromtin SL®, Siemens Healthineers, Germany), Prothrombin time (Dade Innovin®, Siemens Healthineers, Germany), Fibrinogen level (Test Thrombin® reagent, Siemens Healthineers, Germany) and Factor XIII (Berichrom® F XII, Siemens Healthineers, Germany) on a Sysmex CS5100 analyzer. VWF antigen and VWF activity and their ratio were quantified using the INNOVANCE® VWF Ac and the VWF Ag reagent (both Siemens Healthineers, Germany).

To assess platelet function PFA-200 and light transmission aggregometry (LTA) were performed. The PFA-200 (Siemens Healthineers, Germany) assay was carried out following the manufacturer's instructions using Col/ADP and Col/Epi cartridges. LTA was performed on an APACT 4.0 aggregometer (BioMedical Technologies, Ahrensburg, Germany) according to the SSC/ISTH guidelines.

The full postoperative coagulation work-up was available in 10 out of the 17 cases with CIPHEI. None of these 10 CIPHEI patients showed evidence of a coagulation factor deficiency or von Willebrand disease. Two patients displayed abnormalities in tests of primary hemostasis: One patient showed prolonged PFA-200 Col/Epi closure time and consistently a platelet function defect related to NSAID intake (Ibuprofene) which, however, was started postoperatively. One patient had an LTA result suggestive of drug-induced platelet dysfunction due to serotonin–norepinephrine reuptake inhibitor treatment.

S5 Characteristics of the overall sample

Table S5. Characteristics of the overall sample.

				Procedure-related variables									Patient-related variables						
DBS procedures				CMM electrode type			DBS electrode type				Laterality		Age (years)			Gender		aHTN	
Invasivity	Disease	Target	n	None	FHC	AO	MT-O	BS-O	BS-D	AN-D	Bi	Uni	Md	IQR	Range	M	F	w/	w/o
Low	DYS	GPI	56	56			30	11	15		52	4	53.1	36.9-63.3	1.6-77.9	29	27	12	44
	EPI	ANT	7	7			7		0		7		35.4	28.6-42.4	20.7-45.8	4	3	1	6
	PAI	VCP+PAG	8	8			4		4			8	66.2	49.0-69.9	39.3-77.7	4	4	4	4
		Other	3	3			1		2		2	1	65.4	56.8-69.8	48.2-74.3	1	2	2	1
	PD	GPI	4	4			3		1		4		74.8	72.6-75.8	66.6-78.0	2	2	3	1
		STN	8	8			4		4		7	1*	57.4	48.6-67.7	36.5-76.9	7	1	3	5
		STN/DRT	18	18				18	0		17	1	68.3	61.3-74.4	53.6-77.3	12	6	7	11
		VIM	10	10			5		5		5	5	75.8	73.3-80.2	62.3-82.0	8	2	5	5
	PSY	MFB	2	2				1	1		2		43.6	37.7-49.5	31.8-55.3	1	1	1	1
		Other	1	1			1					1	36.6			1			1
	TRE	VIM	61	61			45	2	14		51	10	67.9	59.4-73.7	36.5-82.0	27	34	30	31
		Other	1	1					1			1	38.2			1			1
Medium: CMM	PAI	VCP+PAG	5		3	2	3		2			5	63.2	62.0-68.6	32.2-72.4	4	1	2	3
	PD	VIM	3		3		3				1	2	67.8	65.5-70.4	63.3-73.0	3		2	1
	TRE	VIM	15		8	7	9		6		13	2	66.1	53.1-70.1	34.7-75.6	11	4	6	9

Table S5. Characteristics of the overall sample (continued).

				Procedure-related variables									Patient-related variables						
				CMM electrode type			DBS electrode type				Laterality		Age (years)			Gender		aHTN	
Invasivity	Disease	Target	n	None	FHC	AO	MT-O	BS-O	BS-D	AN-D	Bi	Uni	Md	IQR	Range	M	F	w/	w/o
High: CMM+MER	DYS	GPI	11		5	6	6	1	4		11		44.9	30.5-60.0	10.3-65.3	5	6	3	8
		STN	1			1	1				1		41.0				1		1
	PD	GPI	1		1		1				1		83.8			1		1	
		STN	170		72	98	42	13	113	2	166	4[#]	62.6	55.3-68.1	38.4-77.8	109	61	46	124
	PSY	MFB	50		6	44	2		48		50		42.4	33.7-51.3	25.8-69.7	28	22	12	38
	TRE	Other	1		1		1					1	60.2				1	1	
Overall			436	179	99	158	168	46	220	2	390	46	60.7	50.4-68.5	1.6-83.8	258	178	141	295

Abbreviations: DBS, deep brain stimulation. Invasivity: CMM, combined micro/macroelectrodes; MER, microelectrode recording. Please refer to Table 1 of the main article for the specification of CMM (FHC, AO) and DBS electrode types (MT-O, BS-O, BS-D, AN-D). Diseases: DYS, dystonia; EPI, epilepsy; PAI, chronic pain; PD, Parkinson's disease; PSY, psychiatric disease (i.e. therapy-refractory depression or obsessive compulsive disorder); TRE, non-Parkinsonian Tremor. Targets: ANT, anterior nucleus of the thalamus; DRT, dentato-rubrothalamic tract; GPI, globus pallidus internus; MFB, medial forebrain bundle; PAG periaqueductal gray; STN, subthalamic nucleus; VIM, ventral intermediate nucleus of the thalamus; VCP, parvocellular part of ventrocaudal nucleus of the thalamus. Laterality refers to laterality of implantation: Bi, bilateral; Uni, unilateral. Age: Md, median; IQR, interquartile range. Gender: M, male; F, female. aHTN, arterial hypertension: w/, with; w/o, without. Markers: * unilateral revision of a DBS electrode; [#] one unilateral revision of a DBS electrode and three aborted surgeries due to CIPHEI with intraoperative symptom onset

S6 Explorative Analysis (Left-Hemispheric Procedures)

Bivariable Analyses

Qualitative observations drawn from Table 2 (main text) were further consolidated in bivariable risk analyses taking the underlying base rates into account. To this end, age was binarized (≥60 years) with younger age as reference, gender was set to females as reference (i.e. tested for effects of male gender), type of CMM was set to none as reference (i.e. tested for effects of using AO or FHC CMM); and type of DBS electrode was binarized (BS-D vs. other) given that CIPHEIs were only observed for this type of implant. Level of invasivity was also binarized depending on the outcome variable: For analysis of CIPHEI at any point of the trajectory, medium and high levels of invasivity (i.e. the use of CMM irrespective of MER) was contrasted with low invasivity (no use of CMM) as reference; for analysis of deep CIPHEI, high levels of invasivity (CMM with MER) was contrasted with medium and low levels of invasivity (i.e. CMM without MER or no CMM at all) as reference.

Significantly increased odds ratios (or trends thereof) reflecting higher risks for CIPHEI at any point of the trajectory (or trends thereof) were revealed for patient age ≥60 years, male gender, use of CMM (irrespective of MER, i.e., medium or high invasivity), and for use of AO or FHC CMM and implantation of BS-D electrodes whereas comorbid arterial hypertension was not associated with increased odd ratios (Table S6-1). When focussing on deep CIPHEI, bivariable analysis yielded only a trend for increased odds ratios for use of FHC CMM but no significant effects for any of the risk variables, although this might rather reflect an issue of test power due to the fewer occasions of deep bleedings.

Table S6-1. Bivariable model estimates for left-hemispheric CIPHEI (exploration analysis).

Dependent variable	Risk factor	OR [95% CI]	p-value
Any CIPHEI along trajectory	Age (≥ 60 years)	25.75 [3.37-Inf]	0.02498
	Gender (male)	5.35 [1.26-140.86]	0.05563
	aHTN (w/)	1.65 [0.47-5.15]	0.38114
	Use of CMM	17.45 [2.28-Inf]	0.04861
	CMM electrode type (FHC)	12.60 [1.22-Inf]	0.09606
	CMM electrode type (AO)	21.59 [2.72-Inf]	0.03531
	DBS electrode type (BS-D)	25.25 [3.31-Inf]	0.02588
Deep CIPHEI	Age (≥ 60 years)	8.91 [0.95-Inf]	0.14409
	Gender (male)	1.58 [0.26-41.11]	0.64374
	aHTN (w/)	0.95 [0.04-5.85]	0.95749
	Use of CMM/MER	7.34 [0.78-Inf]	0.18333
	CMM electrode type (FHC)	12.60 [1.22-Inf]	0.09606
	CMM electrode type (AO)	3.23 [0.18-Inf]	0.47479
	DBS electrode type (BS-D)	8.74 [0.93-Inf]	0.14769

Abbreviations: aHTN, history of arterial hypertension; CI, confidence interval; CIPHEI, cerebral intraparenchymal hemorrhage due to electrode implantation; CMM, combined micro/macroelectrodes; DBS, deep brain stimulation; MER, microelectrode recording; OR, odds ratio. Please refer to Table 1 (main text) for the specification of CMM and DBS electrode types. Due to sparse sampling of CIPHEI, for some terms the upper bound of the OR's confidence interval was only estimable with large uncertainty (Inf, infinity).

Given that increased odds ratios for using a specific type of CMM implicitly reflected also the use of CMM at all, we further tested whether the two different types (AO, FHC) significantly differed in their specific risks but found no indications thereof in the bivariable analyses (CIPHEI at any point of the trajectory, p = 0.40157; deep CIPHEI, p = 0.16879). Further note that corresponding to the above indicated clustering of specific risk variables in cases with CIPHEI, several bivariable analyses manifested perfect separations (i.e., age ≥60 years, medium/high levels of invasivity [CMM/MER], use of BS-D electrodes).

Multivariable Analyses - Additive Combination of Risk Factors

Analyses are reported in the main text. For CIPHEI at any point of the trajectory, note that specification of the CMM types resulted in a slightly better model fit (AIC, 77.729 at Step 2; Supplementary Table S6-2) than simply modeling the use of CMM irrespective of MER (AIC, 78.318). Likewise, for deep CIPHEI, specification of CMM electrode types resulted in a far better model fit (AIC, 40.203 at Step 1; Supplementary Table S6-3) than the mere specification of CMM with MER (AIC, 47.142).

Table S6-2. Multivariable additive modeling for left-hemispheric CIPHEI (exploration analysis).

Dependent variable	Step	Additive risk factor combination	AIC
Any CIPHEI along trajectory	1	Full model: Age+Gender+aHTN+CMM+CMM electrode type+DBS electrode type	79.544
		... ex Age (Age ≥ 60 years: yes vs. no)	94.312
		... ex Gender (male vs. female)	80.477
		... ex aHTN (w/ vs. w/o)	77.729
		... ex CMM (use of CMM: yes vs. no)	79.544
		... ex CMM electrode type (none vs. FHC vs. AO)	80.232
		... ex DBS electrode type (BS-D vs. other)	87.538
	2	Reduced model: Age+Gender+CMM+CMM electrode type+DBS electrode type	77.729
		... ex Age (Age ≥ 60 years: yes vs. no)	93.938
		... ex Gender (male vs. female)	78.861
		... ex CMM (use of CMM: yes vs. no)	77.729
		... ex CMM electrode type (none vs. FHC vs. AO)	78.318
		... ex DBS electrode type (BS-D vs. other)	85.545
	3	Reduced model: Age+Gender+CMM electrode type+DBS electrode type	77.729
		... ex Age (Age ≥ 60 years: yes vs. no)	93.938
		... ex Gender (male vs. female)	78.861
		... ex CMM electrode type (none vs. FHC vs. AO)	82.595
		... ex DBS electrode type (BS-D vs. other)	85.545

Abbreviations: aHTN, history of arterial hypertension; CIPHEI, cerebral intraparenchymal hemorrhage due to electrode implantation; CMM, combined micro/macroelectrodes; DBS, deep brain stimulation. Please refer to Table 1 (main text) for the specification of CMM and DBS electrode types.

Table S6-3. Multivariable additive modeling for left-hemispheric CIPHEI (exploration analysis).

Dependent variable	Step	Additive risk factor combination	AIC
Deep CIPHEI	1	Full model: Age+Gender+aHTN+CMM/MER+CMM electr. type+DBS electrode type	43.095
		... ex Age (Age ≥ 60 years: yes vs. no)	48.505
		... ex Gender (male vs. female)	40.649
		... ex aHTN (w/ vs. w/o)	40.567
		... ex CMM/MER (use of CMM/MER: yes vs. no)	40.203
		... ex CMM electrode type (none vs. FHC vs. AO)	47.142
		... ex DBS electrode type (BS-D vs. other)	49.154
	2	Reduced model: Age+Gender+aHTN+CMM electrode type+DBS electrode type	40.203
		... ex Age (Age ≥ 60 years: yes vs. no)	45.574
		... ex Gender (male vs. female)	37.769
		... ex aHTN (w/ vs. w/o)	37.700
		... ex CMM electrode type (none vs. FHC vs. AO)	46.904
		... ex DBS electrode type (BS-D vs. other)	47.056
	3	Reduced model: Age+Gender+CMM electrode type+DBS electrode type	37.700
		... ex Age (Age ≥ 60 years: yes vs. no)	43.332
		... ex Gender (male vs. female)	35.324
		... ex CMM electrode type (none vs. FHC vs. AO)	44.975
		... ex DBS electrode type (BS-D vs. other)	44.830
	4	Reduced model: Age+CMM electrode type+DBS electrode type	35.324
		... ex Age (Age ≥ 60 years: yes vs. no)	41.075
		... ex CMM electrode type (none vs. FHC vs. AO)	42.714
		... ex DBS electrode type (BS-D vs. other)	42.913

Abbreviations: aHTN, history of arterial hypertension; CIPHEI, cerebral intraparenchymal hemorrhage due to electrode implantation; CMM, combined micro/macroelectrodes; DBS, deep brain stimulation; MER, microelectrode recording. Please refer to Table 1 (main text) for the specification of CMM and DBS electrode types.

Multivariable Analyses - Non-additive Combination of Risk Factors

Analyses are reported in the main text. In addition, for CIPHEI at any point of the trajectory, inspecting the odds in Supplementary Table S6-4 indicated that exclusion of male gender in the third, penultimate step was not driven by a further increase of the odds for CIPHEI in the patients featuring the respective risk combinations (11/48 for step 3 reduced model vs. 12/69 for step 4 reduced model) but rather by the decrease of the odds in the patients not featuring the risk combination (1/356 for step 3 reduced model vs. 0/335 for step 4 reduced model). Further given that the latter phenomenon also reflected the issue of a complete separation and division by zero, the resulting odds ratios as well as the evaluations of the model fit can only be approximated (as done here using the approach by Kosmidis & Firth [24]). A potentially relevant non-additive role for male gender can hence not be ruled out.

For deep CIPHEI, the potential role of gender was again borderline (Supplementary Table S6-5), as it was excluded based on a quasi-complete separation in the patients not featuring the risk combination (2/408 for step 2 reduced model vs. 1/406 for step 3 reduced model), whereas the odds in the risk patients remained constant (2/4 for step 2 reduced model vs. 3/6 for step 3 reduced model).

Table S6-4. Multivariable non-additive modeling for left-hemispheric CIPHEI (exploration analysis).

Dependent variable	Step	Multiplicative risk factor combination	Odds	OR [95% CI]	p-value	AIC
Any CIPHEI along trajectory	1	Full model: Age × Gender × aHTN × CMM × CMM electrode type × DBS electrode type	1/1 vs. 11/403	35.09 [1.39 - 968.13]	0.01385	108.313
		... ex Age (Age ≥ 60 years: yes vs. no)	1/2 vs. 11/402	21.00 [0.81 - 205.41]	0.01328	109.328
		... ex Gender (male vs. female)	1/1 vs. 11/403	35.09 [1.39 - 968.13]	0.01385	108.313
		... ex aHTN (w/ vs. w/o)	2/4 vs. 10/400	21.19 [2.57 - 115.96]	0.00077	105.701
		... ex CMM (use of CMM: yes vs. no)	1/1 vs. 11/403	35.09 [1.39 - 968.13]	0.01385	108.313
		... ex CMM electrode type (FHC vs. other)	5/16 vs. 7/388	17.27 [4.70 - 60.55]	0.00001	97.438
		... ex DBS electrode type (BS-D vs. other)	1/7 vs. 11/397	6.91 [0.26 - 35.89]	0.05196	111.351
	2	Reduced model: Age × Gender × aHTN × CMM × DBS electrode type	5/16 vs. 7/388	17.27 [4.70 - 60.55]	0.00001	97.438
		... ex Age (Age ≥ 60 years: yes vs. no)	5/25 vs. 7/379	10.91 [3.02 - 36.42]	0.00008	101.101
		... ex Gender (male vs. female)	5/23 vs. 7/381	11.90 [3.29 - 40.04]	0.00005	100.416
		... ex aHTN (w/ vs. w/o)	11/48 vs. 1/356	56.36 [13.04 - 1507.45]	0.00001	74.711
		... ex CMM (use of CMM: yes vs. no)	5/25 vs. 7/379	10.91 [3.02 - 36.42]	0.00008	101.101
		... ex DBS electrode type (BS-D vs. other)	5/24 vs. 7/380	11.39 [3.15 - 38.15]	0.00006	100.766
	3	Reduced model: Age × Gender × CMM × DBS electrode type	11/48 vs. 1/356	56.36 [13.04 - 1507.45]	0.00001	74.711
		... ex Age (Age ≥ 60 years: yes vs. no)	11/102 vs. 1/302	22.63 [5.32 - 598.78]	0.00038	89.768
		... ex Gender (male vs. female)	12/69 vs. 0/335	120.68 [15.66 - Inf]	0.00095	72.966
		... ex CMM (use of CMM: yes vs. no)	11/66 vs. 1/338	39.03 [9.10 - 1038.16]	0.00003	81.007
		... ex DBS electrode type (BS-D vs. other)	11/69 vs. 1/335	37.01 [8.64 - 983.91]	0.00004	81.895
	4	Reduced model: Age × CMM × DBS electrode type	12/69 vs. 0/335	120.68 [15.66 - Inf]	0.00095	72.966
		... ex Age (Age ≥ 60 years: yes vs. no)	12/158 vs. 0/246	38.88 [5.088 - Inf]	0.01152	91.767
		... ex CMM (use of CMM: yes vs. no)	12/101 vs. 0/303	74.75 [9.75 - Inf]	0.00291	81.509
		... ex DBS electrode type (BS-D vs. other)	12/108 vs. 0/296	68.32 [8.92 - Inf]	0.00356	83.032

Abbreviations: aHTN, history of arterial hypertension; CI, confidence interval. CIPHEI, cerebral intraparenchymal hemorrhage due to electrode implantation; CMM, combined micro/macroelectrodes; DBS, deep brain stimulation; OR, odds ratio. Please refer to Table 1 (main text) for the specification of CMM and DBS electrode types. Due to sparse sampling of CIPHEI, for some terms the upper bound of the OR's confidence interval was only estimable with large uncertainty (Inf, infinity).

Table S6-5. Multivariable non-additive modeling for left-hemispheric CIPHEI (exploration analysis).

Dependent variable	Step	Multiplicative risk factor combination	Odds	OR [95% CI]	p-value	AIC
Deep CIPHEI	1	Full model: Age × Gender × aHTN × CMM × CMM electrode type × DBS electrode type	1/1 vs. 3/411	117.57 [110.86 - Inf]	0.00163	42.388
		... ex Age (Age ≥ 60 years: yes vs. no)	1/2 vs. 3/410	70.37 [2.76 - 957.42]	0.00115	43.442
		... ex Gender (male vs. female)	1/1 vs. 3/411	117.57 [4.71 - 4114.51]	0.00163	42.388
		... ex aHTN (w/ vs. w/o)	2/4 vs. 2/408	90.78 [10.29 - 1060.87]	0.00002	37.041
		... ex CMM/MER (use of CMM/MER: yes vs. no)	1/1 vs. 3/411	117.57 [4.71 - 4114.51]	0.00163	42.388
		... ex CMM electrode type (FHC vs. other)	1/17 vs. 3/395	9.69 [0.37 - 64.27]	0.02691	47.252
		... ex DBS electrode type (BS-D vs. other)	1/5 vs. 3/407	31.75 [1.23 - 261.20]	0.00223	45.049
	2	Reduced model: Age × Gender × CMM × CMM electrode type × DBS electrode type	2/4 vs. 2/408	90.78 [10.29 - 1060.87]	0.00002	37.041
		... ex Age (Age ≥ 60 years: yes vs. no)	2/16 vs. 2/396	24.03 [2.83 - 217.30]	0.00075	41.896
		... ex Gender (male vs. female)	3/6 vs. 1/406	145.92 [20.93 - 4458.38]	<0.00001	29.671
		... ex CMM/MER (use of CMM/MER: yes vs. no)	2/4 vs. 2/408	90.78 [10.29 - 1060.87]	0.00002	37.041
		... ex CMM electrode type (FHC vs. other)	3/50 vs. 1/362	16.75 [2.72 - 443.68]	0.00447	41.091
		... ex DBS electrode type (BS-D vs. other)	2/19 vs. 2/393	20.18 [2.38 - 180.13]	0.00133	42.521
	3	Reduced model: Age × CMM/MER × CMM electrode type × DBS electrode type	3/6 vs. 1/406	145.92 [20.93 - 4458.38]	<0.00001	29.671
		... ex Age (Age ≥ 60 years: yes vs. no)	3/24 vs. 1/388	37.00 [5.89 - 1000.89]	0.00033	36.998
		... ex CMM/MER (use of CMM/MER: yes vs. no)	3/6 vs. 1/406	145.92 [20.93 - 4458.38]	<0.00001	29.671
		... ex CMM electrode type (FHC vs. other)	4/71 vs. 0/341	42.99 [4.56 - Inf]	0.01213	36.278
		... ex DBS electrode type (BS-D vs. other)	3/35 vs. 1/377	24.81 [4.00 - 662.74]	0.00127	39.101

Abbreviations: aHTN, history of arterial hypertension; CI, confidence interval; CIPHEI, cerebral intraparenchymal hemorrhage due to electrode implantation; CMM, combined micro/macroelectrodes; DBS, deep brain stimulation; MER, microelectrode recording; OR, odds ratio. Please refer to Table 1 (main text) for the specification of CMM and DBS electrode types. Due to sparse sampling of CIPHEI, for some terms the upper or both bound of the OR's confidence interval was only estimable with large uncertainty (Inf, infinity).

Summary of Exploratory Risk Analyses in Left-Hemispheric Procedures

Comparing the applied (simple) additive and non-additive models based on AICs suggested better model fits of the latter for explaining CIPHEI at any point of the trajectory (AICs, 77.729 for step 3 reduced model vs. 72.966 for step 4 reduced model in Supplementary Tables S6-2 and S6-4, respectively) and deep CIPHEI (AICs, 35.324 for step 4 reduced model vs. 29.671 for step 3 reduced model in Supplementary Tables S6-3 and S6-5, respectively).

S7 Cross-Validation Analysis (Right-Hemispheric Procedures)

Bivariable Analyses

Significantly increased odds ratios (or trends thereof) in the bivariable analysis of CIPHEI at any point of the trajectory (Supplementary Table S7-1) were found for age ≥ 60 years, and the use of AO CMM and BS-D DBS electrode types, whereas the use of CMM (irrespective of MER) was marginally approaching a trend for an increased risk. No significantly increased odds ratios were found for deep CIPHEI (Supplementary Table S7-1).

Table S7-1. Bivariable model estimates for right-hemispheric CIPHEI (cross-validation analysis).

Dependent variable	Risk factor	OR [95% CI]	p-value
Any CIPHEI along trajectory	Age (≥ 60 years)	3.43 [0.94-26.98]	0.09020
	Gender (male)	2.43 [0.66-19.12]	0.22196
	aHTN (w/)	1.02 [0.20-3.47]	0.97957
	Use of CMM	4.22 [0.96-110.93]	0.10471
	CMM electrode type (FHC)	2.84 [0.32-74.12]	0.31738
	CMM electrode type (AO)	5.44 [1.18-143.39]	0.06103
	DBS electrode type (BS-D)	3.40 [0.93-26.71]	0.09285
Deep CIPHEI	Age (≥ 60 years)	2.99 [0.55-78.18]	0.24983
	Gender (male)	2.12 [0.39-55.61]	0.42799
	aHTN (w/)	0.19 [0.00-1.72]	0.27130
	Use of CMM/MER	8.71 [0.99-Inf]	0.14490
	CMM electrode type (FHC)	8.58 [0.71-Inf]	0.16820
	CMM electrode type (AO)	7.47 [0.73-Inf]	0.18624
	DBS electrode type (BS-D)	2.96 [0.55-77.41]	0.25414

Abbreviations: aHTN, history of arterial hypertension; CI, confidence interval; CIPHEI, cerebral intraparenchymal hemorrhage due to electrode implantation; CMM, combined micro/macroelectrodes; DBS, deep brain stimulation; MER, microelectrode recording; OR, odds ratio. Please refer to Table 1 (main text) for the specification of CMM and DBS electrode types. Due to sparse sampling of CIPHEI, for some terms the upper bound of the OR's confidence interval was only estimable with large uncertainty (Inf, infinity).

Multivariable Analyses - Additive and Non-additive Combinations of Risk Factors

Final model fits in terms of AICs also suggested the non-additive multiplicative feature combinations over the respective additive models (AICs, 91.678 vs. 85.545 in Supplementary Tables S7-2 and S7-4 for any CIPHEI along trajectory, and 52.473 vs. 48.976 in Supplementary Tables S7-3 and S7-5 for deep CIPHEI, respectively).

Male gender was excluded during backward elimination as in the explorative analyses, but in the cross-validation analyses this could not be attributed to potential separation effects as before (Supplementary Materials, S6).

Table S7-2. Multivariable additive modeling for right-hemispheric CIPHEI (cross-validation analysis).

Dependent variable	Step	Additive risk factor combination	AIC
Any CIPHEI along trajectory	1	Full model: Age+Gender+aHTN+CMM+CMM electrode type+DBS electrode type	97.824
		... ex Age (Age ≥ 60 years: yes vs. no)	99.388
		... ex Gender (male vs. female)	96.579
		... ex aHTN (w/ vs. w/o)	95.652
		... ex CMM (use of CMM: yes vs. no)	97.824
		... ex CMM electrode type (none vs. FHC vs. AO)	95.625
		... ex DBS electrode type (BS-D vs. other)	95.952
	2	Reduced model: Age+Gender+aHTN+CMM+DBS electrode type	95.625
		... ex Age (Age ≥ 60 years: yes vs. no)	97.299
		... ex Gender (male vs. female)	94.430
		... ex aHTN (w/ vs. w/o)	93.461
		... ex CMM (use of CMM: yes vs. no)	95.755
		... ex DBS electrode type (BS-D vs. other)	94.626
	3	Reduced model: Age+Gender+CMM+DBS electrode type	93.461
		... ex Age (Age ≥ 60 years: yes vs. no)	95.142
		... ex Gender (male vs. female)	92.299
		... ex CMM (use of CMM: yes vs. no)	93.703
		... ex DBS electrode type (BS-D vs. other)	92.531
	4	Reduced model: Age+CMM+DBS electrode type	92.299
		... ex Age (Age ≥ 60 years: yes vs. no)	94.371
		... ex CMM (use of CMM: yes vs. no)	92.819
		... ex DBS electrode type (BS-D vs. other)	91.678
	5	Reduced model: Age+CMM	91.678
		... ex Age (Age ≥ 60 years: yes vs. no)	93.713
		... ex CMM (use of CMM: yes vs. no)	94.231

Abbreviations:aHTN, history of arterial hypertension; CIPHEI, cerebral intraparenchymal hemorrhage due to electrode implantation; CMM, combined micro/macroelectrodes; DBS, deep brain stimulation. Please refer to Table 1 (main text) for the specification of CMM and DBS electrode types.

Table S7-3. Multivariable additive modeling for right-hemispheric CIPHEI (cross-validation analysis).

Dependent variable	Step	Additive risk factor combination	AIC
Deep CIPHEI	1	Full model: Age+Gender+aHTN+CMM/MER+CMM electr. type+DBS electrode type	61.504
		... ex Age (Age ≥ 60 years: yes vs. no)	62.222
		... ex Gender (male vs. female)	59.688
		... ex aHTN (w/ vs. w/o)	61.534
		... ex CMM/MER (use of CMM/MER: yes vs. no)	58.982
		... ex CMM electrode type (none vs. FHC vs. AO)	56.430
		... ex DBS electrode type (BS-D vs. other)	59.466
	2	Reduced model: Age+Gender+aHTN+CMM/MER+DBS electrode type	56.430
		... ex Age (Age ≥ 60 years: yes vs. no)	57.013
		... ex Gender (male vs. female)	54.628
		... ex aHTN (w/ vs. w/o)	56.820
		... ex CMM/MER (use of CMM/MER: yes vs. no)	57.173
		... ex DBS electrode type (BS-D vs. other)	54.145
	3	Reduced model: Age+Gender+aHTN+CMM/MER	54.145
		... ex Age (Age ≥ 60 years: yes vs. no)	54.770
		... ex Gender (male vs. female)	52.473
		... ex aHTN (w/ vs. w/o)	54.632
		... ex CMM/MER (use of CMM/MER: yes vs. no)	55.936
	4	Reduced model: Age+aHTN+CMM/MER	52.473
		... ex Age (Age ≥ 60 years: yes vs. no)	53.238
		... ex aHTN (w/ vs. w/o)	53.045
		... ex CMM/MER (use of CMM/MER: yes vs. no)	54.255

Abbreviations: aHTN, history of arterial hypertension; CIPHEI, cerebral intraparenchymal hemorrhage due to electrode implantation; CMM, combined micro/macroelectrodes; DBS, deep brain stimulation; MER, microelectrode recording. Please refer to Table 1 (main text) for the specification of CMM and DBS electrode types.

Table S7-4. Multivariable non-additive modeling for right-hemispheric CIPHEI (cross-validation analysis).

Dependent variable	Step	Multiplicative risk factor combination	Odds	OR [95% CI]	p-value	AIC
Any CIPHEI along trajectory	1	Full model: Age × Gender × aHTN × CMM × CMM electrode type × DBS electrode type	0/1 vs. 10/397	0 [n.e. - n.e.]	0	97.899
		... ex Age (Age ≥ 60 years: yes vs. no)	0/2 vs. 10/396	0 [n.e. - n.e.]	0	97.849
		... ex Gender (male vs. female)	0/1 vs. 10/397	0 [n.e. - n.e.]	0	97.899
		... ex aHTN (w/ vs. w/o)	1/4 vs. 9/394	13.84 [0.53 - 85.32]	0.01533	95.327
		... ex CMM (use of CMM: yes vs. no)	0/1 vs. 10/397	0 [n.e. - n.e.]	0	97.899
		... ex CMM electrode type (FHC vs. other)	2/17 vs. 8/381	6.41 [0.81 - 25.49]	0.01661	94.866
		... ex DBS electrode type (BS-D vs. other)	0/9 vs. 10/389	0 [n.e. - n.e.]	0	97.497
	2	Reduced model: Age × Gender × aHTN × CMM × DBS electrode type	2/17 vs. 8/381	6.41 [0.81 - 25.49]	0.01661	94.866
		... ex Age (Age ≥ 60 years: yes vs. no)	2/26 vs. 8/372	4.13 [0.52 - 15.91]	0.06102	96.122
		... ex Gender (male vs. female)	2/24 vs. 8/374	4.50 [0.57 - 17.39]	0.04810	95.897
		... ex aHTN (w/ vs. w/o)	6/50 vs. 4/348	9.97 [2.88 - 42.04]	0.00029	85.991
		... ex CMM (use of CMM: yes vs. no)	2/24 vs. 8/374	4.50 [0.57 - 17.39]	0.04810	95.897
		... ex DBS electrode type (BS-D vs. other)	2/27 vs. 8/371	3.97 [0.50 - 15.26]	0.06815	96.226
	3	Reduced model: Age × Gender × CMM × DBS electrode type	6/50 vs. 4/348	9.97 [2.88 - 42.04]	0.00029	85.991
		... ex Age (Age ≥ 60 years: yes vs. no)	6/104 vs. 4/294	4.07 [1.19 - 16.86]	0.002482	93.092
		... ex Gender (male vs. female)	7/72 vs. 3/326	9.65 [2.80 - 49.92]	0.00062	85.545
		... ex CMM (use of CMM: yes vs. no)	6/67 vs. 4/331	7.09 [2.06 - 29.65]	0.00187	88.937
		... ex DBS electrode type (BS-D vs. other)	6/74 vs. 4/324	6.29 [1.83 - 26.23]	0.00344	89.912
	4	Reduced model: Age × CMM × DBS electrode type	7/72 vs. 3/326	9.65 [2.80 - 49.92]	0.00062	85.545
		... ex Age (Age ≥ 60 years: yes vs. no)	8/159 vs. 2/239	5.11 [1.39 - 40.17]	0.02520	91.507
		... ex CMM (use of CMM: yes vs. no)	7/101 vs. 3/297	6.28 [1.84 - 32.29]	0.00531	89.544
		... ex DBS electrode type (BS-D vs. other)	7/114 vs. 3/284	5.32 [1.56 - 27.33]	0.01109	90.920

Abbreviations: aHTN, history of arterial hypertension; CI, confidence interval; CIPHEI, cerebral intraparenchymal hemorrhage due to electrode implantation; CMM, combined micro/macroelectrodes; DBS, deep brain stimulation; OR, odds ratio. Please refer to Table 1 (main text) for the specification of CMM and DBS electrode types. Due to sparse sampling of CIPHEI, for some terms the bounds of the OR's confidence interval were not estimable (n.e.).

Table S7-5. Multivariable non-additive modeling for right-hemispheric CIPHEI (cross-validation analysis).

Dependent variable	Step	Multiplicative risk factor combination	Odds	OR [95% CI]	p-value	AIC
Deep CIPHEI	1	Full model: Age × Gender × aHTN × CMM × CMM electrode type × DBS electrode type	0/1 vs. 5/402	0 [n.e. - n.e.]	0	57.977
		... ex Age (Age ≥ 60 years: yes vs. no)	0/2 vs. 5/401	0 [n.e. - n.e.]	0	57.952
		... ex Gender (male vs. female)	0/1 vs. 5/402	0 [n.e. - n.e.]	0	57.977
		... ex aHTN (w/ vs. w/o)	1/4 vs. 4/399	29.59 [1.14 - 227.42]	0.00288	53.991
		... ex CMM/MER (use of CMM/MER: yes vs. no)	0/1 vs. 5/402	0 [n.e. - n.e.]	0	57.977
		... ex CMM electrode type (FHC vs. other)	0/16 vs. 5/387	0 [n.e. - n.e.]	0	57.599
		... ex DBS electrode type (BS-D vs. other)	0/5 vs. 5/398	0 [n.e. - n.e.]	0	57.878
	2	Reduced model: Age × Gender × CMM × CMM electrode type × DBS electrode type	1/4 vs. 4/399	29.59 [3.14 - Inf]	0.00288	53.991
		... ex Age (Age ≥ 60 years: yes vs. no)	1/16 vs. 4/387	7.83 [0.30 - 45.26]	0.03916	56.428
		... ex Gender (male vs. female)	1/7 vs. 4/396	17.62 [0.68 - 112.87]	0.00684	54.993
		... ex CMM/MER (use of CMM/MER: yes vs. no)	1/4 vs. 4/399	29.59 [1.14 - 227.42]	0.00288	53.991
		... ex CMM electrode type (FHC vs. other)	3/48 vs. 2/355	10.26 [1.80 - 85.83]	0.00594	51.713
		... ex DBS electrode type (BS-D vs. other)	1/19 vs. 4/384	6.57 [0.25 - 37.55]	0.05707	56.706
	3	Reduced model: Age × Gender × CMM × DBS electrode type	3/48 vs. 2/355	10.26 [6.03 - Inf]	0.00594	51.713
		... ex Age (Age ≥ 60 years: yes vs. no)	3/101 vs. 2/302	4.17 [0.73 - 34.41]	0.08844	55.443
		... ex Gender (male vs. female)	4/70 vs. 1/333	14.19 [2.60 - 375.07]	0.00546	48.976
		... ex CMM/MER (use of CMM/MER: yes vs. no)	3/70 vs. 2/333	6.62 [1.16 - 54.93]	0.02468	53.668
		... ex DBS electrode type (BS-D vs. other)	3/65 vs. 2/338	7.24 [1.27 - 60.09]	0.01881	53.292
	4	Reduced model: Age × CMM × DBS electrode type	4/70 vs. 1/333	14.19 [13.48 - Inf]	0.00546	48.976
		... ex Age (Age ≥ 60 years: yes vs. no)	4/157 vs. 1/246	4.70 [0.87 - 123.14]	0.10371	54.715
		... ex CMM/MER (use of CMM/MER: yes vs. no)	4/104 vs. 1/299	8.60 [1.58 - 226.21]	0.02381	51.858
		... ex DBS electrode type (BS-D vs. other)	4/103 vs. 1/300	8.71 [1.60 - 229.18]	0.02299	51.789

Abbreviations: aHTN, history of arterial hypertension; CI, confidence interval; CIPHEI, cerebral intraparenchymal hemorrhage due to electrode implantation; CMM, combined micro/macroelectrodes; DBS, deep brain stimulation; MER, microelectrode recording; OR, odds ratio. Please refer to Table 1 (main text) for the specification of CMM and DBS electrode types. Due to sparse sampling of CIPHEI, for some terms the bounds of the OR's confidence interval were not estimable (n.e.) or only with large uncertainty (Inf, infinity).

S8 Additional Control Analyses

Partial dependency between the exploration and cross-validation samples

Given the occurence of bilateral CIPHEIs and, as a result, the partial overlap between CIPHEI cases in the left-hemispheric exploration sample and right-hemispheric cross-validation sample, we repeated the cross-validation analyses for CIPHEI at any point of the trajectory after excluding cases with bilateral CIPHEI events (Supplementary Table S8-1).

Table S8-1. Characteristics of cases with right-hemispheric CIPHEI (excl. bilateral events).

CIPHEI					Procedure-related variables			Patient-related variables		
≤25 mm	>25 mm	Invasivity	Diagnosis	Target	CMM Electrode	DBS Electrode	Laterality	Age (years)	Gender	aHTN
	x	low	TRE	VIM	None	MT-O	Bilateral	79.3	M	w/
	x	high	PSY	MFB	AO	BS-D	Bilateral	57.5	F	w/o
x		high	DYS	GPI	FHC	BS-O	Bilateral	28.4	M	w/o
x		high	PD	STN	AO	BS-D	Bilateral	62.7	M	w/o
x		high	PD	STN	AO	BS-D	Right	70.5	F	w/o

Abbreviations: CIPHEI, cerebral intraparenchymal hemorrhage due to electrode implantation. DBS, deep brain stimulation. CMM, combined micro/macroelectrodes. Please refer to Table 1 for the specification of CMM and DBS electrode types. Diagnoses: DYS, dystonia; PD, Parkinson's disease; PSY, psychiatric disease (i.e. therapy-refractory depression or obsessive-compulsive disorder); TRE, non-Parkinsonian Tremor. Targets: GPI, globus pallidus internus; MFB, medial forebrain bundle; STN, subthalamic nucleus; VIM, ventral intermediate nucleus of the thalamus. Laterality refers to laterality of implantation: Unilateral implantations in STN resulted from abortion of surgery due to CIPHEI with intraoperative symptom onset. Gender: M, male; F, female. aHTN, arterial hypertension: w/, with; w/o, without.

Multivariable analysis with backward elimination resulted in the non-additive combination of age ≥60 years, use of CMM (irrespective of MER), and use of BS-D electrodes as risk factors for CIPHEI at any point of the trajectory, but the respective odds ratios failed to reach significance (OR [95% CI] = 3.26 [0.40 - 18.77]; p = 0.15976).

However, given that the age threshold was arbitrarily set to the fixed value of 60 years and that one of the two patients below this threshold almost approached this threshold (Supplementary Table S8-1), we repeated the analysis after adjusting the threshold to 57.5 years to include this patient into the risk combination. Backward elimination again terminated at the non-additive combination of age older 57.5 years, use of CMM (irrespective of MER), and use of BS-D electrodes as risk factors for CIPHEI at any point of the trajectory with increased odd ratios (OR [95% CI] = 5.66 [0.99 - 46.78], p = 0.03902).

Further note in this respect that the case with an age of 28.5 years and an omnidirectional BS-O electrode being most deviant from the non-additive combination of three risk factors put forward here belonged to the very few exceptional cases for which the trajectory had to be planned based on CT images which were distorted by beam hardening artifacts (see Supplement S9 for details). Thus, excluding this case from the analysis might be also justified, which would render the odds ratios of the above non-additive three-factor combination marginally significant (OR [95% CI] = 4.57 [0.54 - 38.75]; p = 0.09388) even without a minor readjustment of the age threshold.

Taken together, although controlling for the partial dependency between the exploration and cross-validation samples due to bilateral hemorrhagic events further exacerbated the sparse sampling issue in the present data, the respective control analyses provide additional support for the relevance of the non-additive risk-factor combination of older age, insertion of CMM (irrespective of MER), and use of BS-D electrodes for CIPHEI at any point of the trajectory.

S9 Further Considerations

Across the two hemispheres and respective subsamples for exploration (left, n=12 events) and cross-validation (right, n=10 events), we observed 22 CIPHEI events (including 5 bilateral events) in 17 CIPHEI cases. Out of these 22 CIPHEI events in 17 CIPHEI cases, a total of 19 events (86.4%) in 14 cases (82.4%) completely conformed to the mutual three-way interaction (or combined presence) of the three risk factors older age (≥60 years), use of CMM/MER, and directional BS-D electrodes (cf. Tables 2 and 4 in the main text).

The remaining three events had alternative aspects that could serve as an explanation for CIPHEI, as follows:

- After bilateral implantation of BS-D electrodes with preceding MER to treat a pharmacotherapy-resistant psychiatric condition in a 57-year-old female a right-hemispheric delayed CIPHEI was encountered in a FDG-PET/CT 7 days postoperatively that was not present in the CT directly obtained after surgery. This incident reflects that asymptomatic delayed CIPHEI can be detected by chance due to (additional) imaging after the day of surgery as discussed in the limitations. Furthermore, 57 years is very close to our arbitrary threshold of 60 years.
- The postoperative CT in a 79-year-old male with essential tremor who had received a re-implantation (after explantation due to an infection) of MT-O electrodes after testing with a blunt-tip electrode found a right hemispheric CIPHEI. Postinfectious hardening required sharp pial incision for placement of the blunt-tip electrode, which may explain CIPHEI in this particular case.
- A 28-year-old male with bilateral cochlear implants received bilateral GPI-DBS with BS-O electrodes with preceding MER and showed a right-hemispheric CIPHEI in postoperative CT. Preoperative MRI was not feasible due to the cochlear implants and even the preoperative CT angiography for trajectory planning was distorted by beam hardening artifacts that may have concealed blood vessels on the trajectory.

Although this comparison of cases self-evidently illustrates that CIPHEIs constitute a multi-faceted phenomenon with different potential causes, it also highlights that an overwhelming majority of CIPHEIs in the present data could be attributed to the single pattern of the identified three risk factors in combination.

A striking aspect of the present data comprises the accumulation of bilateral CIPHEI events in one third of CIPHEI cases conforming to the three-way combination of risk factors (5 out of 14), whereas no bilateral CIPHEI events occured in the remaining three CIPHEI cases (see above).

On the one hand, this accumulation of bilateral CIPHEI events hampered the statistical independence of the exploration and the cross-validation data sets splitted by hemisphere and required additional control analyses (Supplement S8). However, on the other hand, given the very rare overall incidence of CIPHEIs and the hence extreme unlikeliness of (random) bilateral events, the observation of bilateral CIPHEIs in even one third of the CIPHEI cases featuring the three-way risk factor combination can be seen as a strong argument for the validity and clinical relevance of the three-way mutual dependence of older age (≥60 years), use of CMM/MER, and directional BS-D electrodes.

References of Supplementary Material

1. Patel DM, Walker HC, Brooks R, Omar N, Ditty B, Guthrie BL. Adverse events associated with deep brain stimulation for movement disorders: analysis of 510 consecutive cases. *Neurosurgery*. 2015;11 Suppl 2:190-199. doi:10.1227/NEU.0000000000000659
2. Doshi PK, Rai N, Das D. Surgical and Hardware Complications of Deep Brain Stimulation-A Single Surgeon Experience of 519 Cases Over 20 Years. *Neuromodulation*. 2022;25(6):895-903. doi:10.1111/ner.13360
3. Fenoy AJ, Simpson RK. Risks of common complications in deep brain stimulation surgery: management and avoidance. *J Neurosurg*. 2014;120(1):132-139. doi:10.3171/2013.10.JNS131225
4. Falowski SM, Ooi YC, Bakay RAE. Long-Term Evaluation of Changes in Operative Technique and Hardware-Related Complications With Deep Brain Stimulation. *Neuromodulation*. 2015;18(8):670-677. doi:10.1111/ner.12335
5. Zrinzo L, Foltynie T, Limousin P, Hariz MI. Reducing hemorrhagic complications in functional neurosurgery: a large case series and systematic literature review. *J Neurosurg*. 2012;116(1):84-94. doi:10.3171/2011.8.JNS101407
6. Yang C, Qiu Y, Wang J, Wu Y, Hu X, Wu X. Intracranial hemorrhage risk factors of deep brain stimulation for Parkinson's disease: a 2-year follow-up study. *J Int Med Res*. 2020;48(5):300060519856747. doi:10.1177/0300060519856747
7. Bullard AJ, Hutchison BC, Lee J, Chestek CA, Patil PG. Estimating risk for future intracranial, fully implanted, modular neuroprosthetic systems: A systematic review of hardware complications in clinical deep brain stimulation and experimental human intracortical arrays. *Neuromodulation*. 2020;23(4):411-426. doi:10.1111/ner.13069
8. Sobstyl M, Aleksandrowicz M, Ząbek M, Pasterski T. Hemorrhagic complications seen on immediate intraprocedural stereotactic computed tomography imaging during deep brain stimulation implantation. *J Neurol Sci*. 2019;400:97-103. doi:10.1016/j.jns.2019.01.033
9. Voges J, Waerzeggers Y, Maarouf M, et al. Deep-brain stimulation: long-term analysis of complications caused by hardware and surgery--experiences from a single centre. *J Neurol Neurosurg Psychiatr*. 2006;77(7):868-872. doi:10.1136/jnnp.2005.081232
10. Tonge M, Ackermans L, Kocabicak E, et al. A detailed analysis of intracerebral hemorrhages in DBS surgeries. *Clin Neurol Neurosurg*. 2015;139:183-187. doi:10.1016/j.clineuro.2015.10.017
11. Wang X, Wang J, Zhao H, et al. Clinical analysis and treatment of symptomatic intracranial hemorrhage after deep brain stimulation surgery. *Br J Neurosurg*. 2017;31(2):217-222. doi:10.1080/02688697.2016.1244252
12. Martin AJ, Starr PA, Ostrem JL, Larson PS. Hemorrhage Detection and Incidence during Magnetic Resonance-Guided Deep Brain Stimulator Implantations. *Stereotact Funct Neurosurg*. 2017;95(5):307-314. doi:10.1159/000479287
13. Kimmelman J, Duckworth K, Ramsay T, Voss T, Ravina B, Emborg ME. Risk of

surgical delivery to deep nuclei: a meta-analysis. *Mov Disord*. 2011;26(8):1415-1421. doi:10.1002/mds.23770

14. Videnovic A, Metman LV. Deep brain stimulation for Parkinson’s disease: prevalence of adverse events and need for standardized reporting. *Mov Disord*. 2008;23(3):343-349. doi:10.1002/mds.21753

15. Kleiner-Fisman G, Herzog J, Fisman DN, et al. Subthalamic nucleus deep brain stimulation: summary and meta-analysis of outcomes. *Mov Disord*. 2006;21 Suppl 14:S290-304. doi:10.1002/mds.20962

16. Voges J, Hilker R, Bötzel K, et al. Thirty days complication rate following surgery performed for deep-brain-stimulation. *Mov Disord*. 2007;22(10):1486-1489. doi:10.1002/mds.21481

17. Park CK, Jung NY, Kim M, Chang JW. Analysis of Delayed Intracerebral Hemorrhage Associated with Deep Brain Stimulation Surgery. *World Neurosurg*. 2017;104:537-544. doi:10.1016/j.wneu.2017.05.075

18. Rughani AI, Hodaie M, Lozano AM. Acute complications of movement disorders surgery: effects of age and comorbidities. *Mov Disord*. 2013;28(12):1661-1667. doi:10.1002/mds.25610

19. Jung I-H, Chang KW, Park SH, Chang WS, Jung HH, Chang JW. Complications After Deep Brain Stimulation: A 21-Year Experience in 426 Patients. *Front Aging Neurosci*. 2022;14:819730. doi:10.3389/fnagi.2022.819730

20. Lachenmayer ML, Mürset M, Antih N, et al. Subthalamic and pallidal deep brain stimulation for Parkinson’s disease-meta-analysis of outcomes. *npj Parkinsons Disease*. 2021;7(1):77. doi:10.1038/s41531-021-00223-5

21. Runge J, Nagel JM, Cassini Ascencao L, et al. Are transventricular approaches associated with increased hemorrhage? A comparative study in a series of 624 deep brain stimulation surgeries. *Oper Neurosurg (Hagerstown)*. 2022;23(2):e108-e113. doi:10.1227/ons.0000000000000275

22. Runge J, Cassini Ascencao L, Blahak C, et al. Deep brain stimulation in patients on chronic antiplatelet or anticoagulation treatment. *Acta Neurochir (Wien)*. 2021;163(10):2825-2831. doi:10.1007/s00701-021-04931-y

23. Sorar M, Hanalioglu S, Kocer B, Eser MT, Comoglu SS, Kertmen H. Experience Reduces Surgical and Hardware-Related Complications of Deep Brain Stimulation Surgery: A Single-Center Study of 181 Patients Operated in Six Years. *Parkinsons Dis*. 2018;2018:3056018. doi:10.1155/2018/3056018

24. Kosmidis I, Firth D. Jeffreys-prior penalty, finiteness and shrinkage in binomial-response generalized linear models. *Biometrika*. 2021;108(1):71-82. doi:10.1093/biomet/asaa052
